# The mutation R107Q alters mtSSB ssDNA compaction ability and binding dynamics

**DOI:** 10.1093/nar/gkae354

**Published:** 2024-04-14

**Authors:** Martial Martucci, Amandine Moretton, Aleix Tarrés-Solé, Virginie Ropars, Louise Lambert, Patrick Vernet, Maria Solà, Maria Falkenberg, Geraldine Farge, Siet van den Wildenberg

**Affiliations:** Université Clermont Auvergne, CNRS, Laboratoire de Physique de Clermont, F-63000 Clermont-Ferrand, France; Université Clermont Auvergne, CNRS, Laboratoire de Physique de Clermont, F-63000 Clermont-Ferrand, France; Structural MitoLab, Molecular Biology Institute Barcelona (IBMB-CSIC), Barcelona Science Park, Barcelona 08028, Spain; Université Paris-Saclay, CEA, CNRS, Institute for Integrative Biology of the Cell (I2BC), 91198 Gif-sur-Yvette, France; Université Clermont Auvergne, CNRS, Laboratoire de Physique de Clermont, F-63000 Clermont-Ferrand, France; Department of Medical Biochemistry and Cell Biology, University of Gothenburg, P.O. Box 440, SE-405 30 Gothenburg, Sweden; Université Clermont Auvergne, CNRS, Laboratoire de Physique de Clermont, F-63000 Clermont-Ferrand, France; Structural MitoLab, Molecular Biology Institute Barcelona (IBMB-CSIC), Barcelona Science Park, Barcelona 08028, Spain; Department of Medical Biochemistry and Cell Biology, University of Gothenburg, P.O. Box 440, SE-405 30 Gothenburg, Sweden; Université Clermont Auvergne, CNRS, Laboratoire de Physique de Clermont, F-63000 Clermont-Ferrand, France; Université Clermont Auvergne, CNRS, Laboratoire de Physique de Clermont, F-63000 Clermont-Ferrand, France; Université Clermont Auvergne, CNRS, IRD, Université Jean Monnet Saint Etienne, LMV, F-63000 Clermont-Ferrand, France

## Abstract

Mitochondrial single-stranded DNA-binding protein (mtSSB) is essential for mitochondrial DNA (mtDNA) replication. Recently, several mtSSB variants have been associated with autosomal dominant mitochondrial optic atrophy and retinal dystrophy. Here, we have studied at the molecular level the functional consequences of one of the most severe mtSSB variants, R107Q. We first studied the oligomeric state of this variant and observed that the mtSSB^R107Q^ mutant forms stable tetramers *in vitro*. On the other hand, we showed, using complementary single-molecule approaches, that mtSSB^R107Q^ displays a lower intramolecular ssDNA compaction ability and a higher ssDNA dissociation rate than the WT protein. Real-time competition experiments for ssDNA-binding showed a marked advantage of mtSSB^WT^ over mtSSB^R107Q^. Combined, these results show that the R107Q mutation significantly impaired the ssDNA-binding and compacting ability of mtSSB, likely by weakening mtSSB ssDNA wrapping efficiency. These features are in line with our molecular modeling of ssDNA on mtSSB showing that the R107Q mutation may destabilize local interactions and results in an electronegative spot that interrupts an ssDNA-interacting-electropositive patch, thus reducing the potential mtSSB-ssDNA interaction sites.

## Graphical Abstract

**Graphical Abstract:**
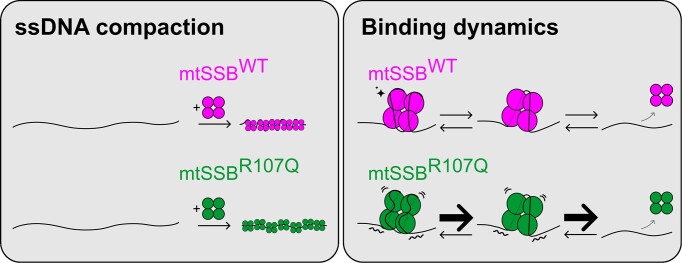

## Introduction

Single-stranded DNA (ssDNA) intermediates are generated during common DNA maintenance processes such as replication, transcription, repair and recombination. Single-stranded DNA binding proteins (SSBs) bind those ssDNA intermediates with high affinity, preventing the formation of secondary structures and protecting them against chemical and enzymatic degradation. The mitochondrial ssDNA-binding protein (mtSSB) is a key actor of mitochondrial DNA (mtDNA) replication. It covers and stabilizes the long stretches of ssDNA formed during mtDNA replication, but also interacts directly or indirectly with the other components of the replication machinery, which includes the heterotrimeric DNA polymerase γ (POLγ) and the replicative DNA helicase TWINKLE (1,2,3,4). Although it seems that there are no physical interactions between mtSSB and POLγ (5), mtSSB is able to stimulate POLγ processivity and the helicase activity of TWINKLE during replication elongation (6). Based on single-molecule assays, a mechanism has been proposed to explain how mtSSB can enhance strand displacement during DNA synthesis by POLγ, in which mtSSB increases the DNA junction destabilization energy and reduces the DNA fork reannealing pressure on POLγ (7). Recently, it was shown that mtSSB plays an additional role in regulating mtDNA replication initiation, by promoting RNA primer formation at the two mtDNA origins of replication thus down-regulating transcription initiation from the light-strand promoter (LSP) (8).

Mammalian mtSSB is a 148 amino acids protein of 16 kDa that possesses a mitochondrial targeting sequence (MTS) (9). While POLγ and TWINKLE are related to phage replicative factors, mtSSB shares with *Escherichia coli* SSB (*Ec*SSB) a conserved N-terminal ssDNA binding domain referred to as OB-fold (oligonucleotide/oligosaccharide binding-fold) (10). In contrast, mtSSB lacks a C-terminal disordered region that is involved in protein–protein interactions in *Ec*SSB and other species (11,12). Depending on the number of OB-fold domains, SSBs can be classified into two groups, simple SSBs which contain one OB-fold domain like mtSSB or *Ec*SSB, and higher order SSBs which contain multiple OB-fold domains like Archaea and Eukarya RPA1, RPA2 and RPA3 (9). Crystallographic studies of human mtSSB showed that the overall structure of the mtSSB resembles that of *Ec*SSB, in which two dimers form a tetramer that is stable in solution (11,13,14). MtSSB binds ssDNA with high affinity and in a sequence independent manner (13). However, unlike *Ec*SSB, which binds ssDNA with negative cooperativity, binding of mtSSB is not cooperative (15).

In *Ec*SSB, several stable or semi-stable DNA binding modes have been described that feature coverage of DNA regions of different lengths, thus the DNA binding site-size is variable, such as (*Ec*SSB)35, (*Ec*SSB)56, (*Ec*SSB)65 and more recently (*Ec*SSB)17, where the numbers indicate the apparent nucleotides bound per tetramer (16). Initial studies showed that in the (*Ec*SSB)35 binding mode, mainly observed at low salt concentrations, the ssDNA only interacts with two protomers of the tetramer, both from the same dimer, as later confirmed by the crystal structure (11,17,18). Further binding of the ssDNA to the second pair of *Ec*SSB protomers occurs with negative cooperativity (15). In the (*Ec*SSB)65 binding mode, found preferentially at high salt concentrations, the same crystallographic studies suggested that two separated regions of the longer ssDNA bind, each of them, a dimer within the tetramer. Thus, the 65-nucleotide DNA wraps around the four *Ec*SSB subunits (11). In both binding modes it was shown that *Ec*SSB diffuses rapidly on ssDNA and can ‘hop or jump’ across long distances of ssDNA via intersegmental transfer (19,20,21,22). In contrast, the related gp2.5 protein from the T7 phage is relatively immobile on ssDNA (23). Similar to *Ec*SSB, two binding modes have also been proposed for mtSSB: a large site binding mode (mtSSB)60 and a shorter site binding mode (mtSSB)30, (10,15,24). The association and dissociation rates of mtSSB have been estimated by equilibrium titrations and stopped-flow kinetic measurements and it was found that the mtSSB tetramer binds ssDNA with a rate of k∼2.10^9^ (M^−1^s^−1^) (15). The binding mode depends on protein, salt, and magnesium concentrations. As the salt concentration is increased, the binding shifts from the short to the larger site mode, correlating with a progressive compaction of the ssDNA towards the larger site mode, and also suggesting different DNA binding mechanisms similar to *Ec*SSB, likely involving binding to one dimer (short site mode) or to all monomers in the tetramer (large site mode). SSB binding modes can also be modulated by interacting proteins. For instance, in bacteria, the DNA helicase PriA, PriC and RecQ were shown to interact with *Ec*SSB and promote the transition from one binding mode to the other (25,26,27). Similarly, when coupled to DNA replication with the bacteriophage Phi29 DNA polymerase, human mtSSB only shows the short site binding mode. The transition from the large to the short site binding modes induced by interacting proteins was suggested to facilitate the access to the buried ssDNA for the replicating proteins (24).

Single-stranded DNA compaction has been visualized for *Ec*SSB using electron microscopy (EM) (28), atomic force microscopy (AFM) (29) and total internal reflection fluorescence (TIRF) microscopy (30). Surprisingly, in the TIRF experiments, this compaction is larger than what would be expected for a simple transition between the two binding modes. The authors proposed that this is due to the ability of *Ec*SSB to interact with distant sites along the ssDNA, either through dimerization of SSB tetramers or through the partial wrapping of distant ssDNA sites on a single SSB tetramer (30). Recently, it was also shown that the T7 gp2.5/ssDNA nucleoprotein complex significantly impacts DNA’s mechanical properties, with a reduction of the DNA’s contour length without modification of the DNA’s persistence length compared to bare ssDNA (23). Additionally, several studies on *Ec*SSB showed that, when high forces are applied on ssDNA, the wrapping mode is destabilized in favor of the binding modes, as they do not require ssDNA to bend and adopt specific conformations as for wrapping. For example, at tensions above 8 pN, only the (*Ec*SSB)17 mode is observed (16,31). mtSSB displays a similar behavior, i.e. a limited ssDNA compaction at high forces, which increases when the force is lowered (24). At even higher forces, SSB proteins detach from ssDNA, and the disassembly force has been estimated around 13 pN for mtSSB and 20 pN for T7 SSB gp2.5 (23,24).

Recently, several mtSSB variants have been reported to cause autosomal dominant mitochondrial optic neuropathy/atrophy and retinal dystrophy with or without mitochondrial DNA depletion. These mutations have been found so far in more than 80 patients across around 20 families and with the development of whole exome sequencing (WES) and whole genome sequencing (WGS), these numbers are rapidly rising (14,32,33,34,35,36). Different hypotheses could explain the molecular mechanisms of the disease: a destabilization of the mtSSB tetramer into dimers or monomers or a perturbation of the interactions of mtSSB with DNA (binding or unbinding) or with other proteins. Some initial studies in patient-derived fibroblasts suggested that the mutations R38Q and R107Q could destabilize mtSSB tetramer formation (14,32). Several of the disease-causing mutants (G40V, N62D, R107Q, E111Q and I132V) have later been studied more in detail in *in vitro* biochemistry experiments. The effect on DNA replication initiation and on the DNA binding capacities were assayed (8). Except for the mutant I132V, all the mutants tested did not affect DNA replication elongation but rather replication initiation, from one or the two mtDNA origins of replication. As for the binding to ssDNA of two different lengths (30 and 100 nt), the three mtSSB variants G40V, N62D and R107Q displayed decreased binding to a short 30-nt ssDNA fragment but a binding pattern similar to that seen with wild-type mtSSB on a longer 100-nt ssDNA fragment, suggesting that the disease-causing mutations could affect the short binding mode of mtSSB. An array of mtSSB variants (E27K, R38Q, G40V, N62D, R107Q, E111Q, I132V and S141N) have also been recently studied using structural analysis predictions (37). These molecular dynamics simulations predict that the mutations do not destabilize the tetrameric structure of mtSSB and do not induce a complete disruption of the DNA binding. Instead, they could lead to more subtle changes in ssDNA binding via local perturbations of specific ssDNA–mtSSB interactions.

Here we have investigated the pathogenesis mechanisms of mtSSB^R107Q^, which presents one of the most severe phenotypes among all the mtSSB disease-causing mutants discovered. We first assayed the oligomeric state of the mtSSB^R107Q^ using mass photometry. We then visualized ssDNA–mtSSB interactions and quantified ssDNA compaction driven by mtSSB using single-molecule fluorescence microscopy and acoustic force spectroscopy. We also determined disassembly and assembly rates. Importantly, we have visualized in real-time the competition between mtSSB^WT^ and mtSSB^R107Q^ for ssDNA. Based on these results and on a structural modeling of ssDNA-wrapped mtSSB^R107Q^, we propose a model to explain the molecular mechanisms associated with the disease phenotype, in which the R107Q mutation alters the wrapping and compacting abilities of mtSSB.

## Material and methods

### Proteins purification and labelling

For fluorescent labelling, the DNA sequence of mtSSB lacking the mitochondrial targeting sequence (aa 1–16) and containing a N-terminal His-tag and a C-terminal cysteine residue was cloned in pET9a expression vector (Novagen). R107Q mutation was introduced using the QuickChange Lightning site-directed mutagenesis kit (Agilent Technologies, Santa Clara, CA) and confirmed by sequencing (Eurofins MWG Operon). mtSSB^WT^ and mtSSB^R107Q^ were expressed in *E. coli* pLys-Rosetta cells in LB medium containing 50 μg/ml kanamycin and 34 μg/ml chloramphenicol. The protein expression was induced by adding 0.5 mM IPTG at OD600 ∼0.6 and the cells were grown at 16°C with shaking overnight before harvesting. The proteins were first purified with HIS-Select® Nickel Affinity Gel (Sigma-Aldrich). The extract was added to 2 ml of the resin equilibrated in buffer 1 (25 mM Tris–HCl, pH 8.0, 0.4 M NaCl, 10% glycerol, 10 mM β-mercaptoethanol and 1X protease inhibitors) containing 10 mM imidazole and incubated for 1 h at 4°C. Ni^2+^-agarose beads were collected by centrifugation, washed once with 15 ml of buffer 1 containing 10 mM imidazole, again collected by centrifugation, and finally loaded into a column. The column was washed with 10 column volumes of buffer A containing 10 mM imidazole and eluted with 15 column volumes of buffer 1 containing 250 mM imidazole. The proteins were further purified using a 5 ml HiTrap heparin HP affinity column (Cytivia) equilibrated in buffer 2 (20 mM Tris pH 8, EDTA 0.5 mM, glycerol 10%, DTT 1 mM, protease inhibitors 1×) containing 0.1 M NaCl. Proteins were eluted with a linear gradient (20 ml) of buffer 2 (0.1–1.2 M NaCl). The mtSSB proteins eluted at a concentration of ∼0.5 M NaCl. The peak fractions were pooled and loaded onto an 1 ml hydroxyapatite column (Foresight™ CHT™ XT Column, BioRad) equilibrated in buffer 3 (glycerol 10%, DTT 1 mM) containing 0.2 M KPO_4_. Proteins were eluted with a linear gradient (10 ml) of buffer 3 (0.2–1.2 M KPO4). Finally, the peak fractions were pooled and loaded onto a gel filtration column (HiLoad® 16/600 Superdex® 200 pg, Cytivia) using buffer 2 (without EDTA) containing 0.1 M NaCl. The construct for mtSSB used in the AFS experiments also encoded the mitochondrial form of the protein without the import signal (1–16 amino acids) but had no affinity tag. They were purified as previously described (6). The purity of all the proteins, estimated by SDS–PAGE with Coomassie blue staining, was >95% (Supplementary Figure S1A).

The proteins were then labelled with Maleimide Alexa Fluor™ 555 dye or Maleimide Alexa Fluor™ 647 dye (Fisher Scientific). Unreacted dye was removed from the sample with size exclusion spin-columns (MicroSpin G-25 columns – Cytiva Amersham). The labelling efficiency was estimated above 90% using Nanodrop One (Thermofisher) and quantification of band intensities on Coomassie blue gels (ImageJ). Protein concentrations were measured with Nanodrop One using an extinction coefficient of 19 940 M^−1^ cm^−1^ and a molecular weight of 16.8 kDa. We checked that the labelling reaction did not affect mtSSB DNA binding ability using electromobility shift-assay and a 22-nucleotide fluorescently-labelled substrate (5′-[ATTO647]GTTAGTTGGGGGGTGACTGTTA-3′) (Supplementary Figure S1B). Briefly, reaction buffer consisted in 20 mM Tris–HCl (pH 7.8), 10 mM MgCl_2_, 0.1 mg/ml BSA, 10 mM DTT, 10% glycerol and 2 mM ATP, supplemented with 50 fmol of ssDNA substrate and indicated final concentrations of mtSSB in a final volume of 15 μl. The reactions were incubated for 10 min at room temperature before separation on 8% native polyacrylamide gels with 0.5× Tris–borate EDTA (TBE) and visualization on Fusion FX gel imaging system (Vilber).

### Mass photometry

Mass photometry experiments were recorded on a Refeyn TwoMP system. Proteins were prepared at 1 μM and diluted prior measurements at a concentration of 100 nM. Data were collected for one minute with the provided Acquire Software. Calibration of the instrument was performed in the same buffer with carbonic anhydrase, BSA, and Urease covering a range of molecular weight from 29 to 816 kDa. Data were analyzed using the provided Discovery analysis software.

### ssDNA constructs

For TIRF experiments, double-stranded Lambda DNA (48502 bp) was biotinylated at the 5′-ends of both strands using Klenow polymerase, as described in (38). The labelled DNA was purified using Illustra MicroSpin S-400 HR columns (GE Healthcare). Biotinylated dsDNA was then denatured using 0.5 M NaOH for 10 min at 37°C as described in (30), then diluted to 0.15 ng/μl final concentration in buffer A (20 mM TrisOAc pH8, 20% sucrose, 20 mM DTT) and kept on ice.

For AFS experiments, DNA substrates were made by PCR amplification (PCR Long Amplification Taq PCR kit, NEB) of a 9 kb fragment of human mitochondrial genome, using a forward primer containing digoxigenin moieties and a reverse primer allowing addition of biotin moieties on the same strand with Klenow polymerase (Forward primer: 5′-DIG-DIG-GCT AAA CCT AGC CCC AAA CC-3′ and reverse primer: 3′-CGG CTC GAG AGG GTT ATG AGA GTA GC-5′). This labelled dsDNA was purified using NucleoSpin Gel and PCR Clean-Up (Macherey-Nagel), and then denatured, diluted at 0.1 ng/ul in cold water and kept on ice.

### TIRF microscopy

The TIRF experimental setup was described in detail in (39). For fluorescent imaging of mtSSB^Alexa555^ and mtSSB^Alexa647^, a 532-nm and 640-nm excitation lasers were used and imaged either on a Nikon (DS-Qi2) wide field camera (compaction assay) or on an EMCCD (iXon 888, ANDOR) camera (dissociation and competition assays). All TIRF experiments were performed in buffer A (20 mM TrisOAc pH8, 20% sucrose, 20 mM DTT) supplemented with the amounts of NaOAc indicated in the text. In all experiments, biotinylated λ ssDNA was mixed with mtSSB^WT^ and/or mtSSB^R107Q^ in 400 μl of buffer A, resulting in a concentration of 0.15 ng/μl for ssDNA and the mtSSB concentrations indicated in the text. The ssDNA–mtSSB complexes formed were anchored on one end to the glass surface and subsequently flow-stretched for imaging.

### Biolayer interferometry (BLI)

Biolayer interferometry experiments were performed using an Octet RED384 device (Fortebio) at 25°C and 1000 rpm shaking rate. Streptavidin-sensors were hydrated in a buffer containing 20 mM HEPES pH 7.6, 100 mM NaCl, 0.1 mM EDTA, 0.5 mM TCEP for 10 min and the baseline was recorded for 2 min. Sensors were then functionalized with a biotinylated poly-dT 30-mer or 60-mer at a concentration of 50 nM until the value of 0.2 nm was reached. Experiments were carried out subsequently in the same buffer supplemented with 0.05% Tween. Association reactions were performed for 300 seconds, and the dissociation for 600 seconds in presence of mtSSB^WT^ or mtSSB^R107Q^ at different concentrations ranging from 0.625 to 5 nM. The data were analyzed using Octet Analysis Studio 12 software and global fitted with a 1:1 binding model.

### Acoustic force spectroscopy (AFS)

For AFS experiments, DNA tethers were made by attaching DNA molecules labelled on one end with digoxigenin and on the other end with biotins between a glass surface and 4.89 μm streptavidin polystyrene beads (Spherotech). A detailed description of the AFS instrumentation (LUMICKS B.V., Amsterdam, the Netherlands) can be found in (39). Flow-cell functionalization was performed with anti-digoxigenin (50 μg/ml, Roche) for 20 min and passivation with PBS containing 2% BSA for at least 30 min. Beads in PBS containing 2% BSA were mixed with the ssDNA substrate diluted in water for 2 min at room temperature before injection in the flow-cell and incubation for 5 min for tethering. Unattached beads were washed first with PBS containing 0.2% BSA and then with 10 mM Tris–HCl pH 7.5, 100 mM NaCl and 0.2% BSA under low force. After selecting the regions of interest, making the look-up tables, and performing the force calibration, force-extension curves of the bare DNA tethers were acquired. These steps with bare DNA constituted the control and were done for each assay. Proteins of interest were then flushed in the flow-cell at a flow rate of 4 μl/min for 2 min, incubated 2 min and rinsed with 10 mM Tris–HCl pH 7.5, 100 mM NaCl and 0.2% BSA for 2 min, and stretching curves were generated. Data acquisition was performed using AFS Tracking G2 and analysis with AFS Analysis G2 software (LUMICKS B.V., Amsterdam, the Netherlands). The force-distance curves were fitted with the extensible Freely Jointed Chain (eFJC) model using MATLAB: L = Lc × coth (F × 2 × Lp(kBT))) – ((kBT)(F × 2 × Lp)1 + (FS)), with L being the distance between the bead and the surface (i.e. the length of the DNA), F the applied force, Lc the contour length of the DNA, Lp the persistence length, k_B_T the product of the Boltzmann constant and the temperature (which is 4.11 pN nm at 25°C), and S the stretch modulus (set at 800 pN for ssDNA (40)).

### Structural superposition of ssDNA onto mtSSB^R107Q^

mtSSB^R107Q^ structure was modeled as explained in (14). Briefly, Arg 107 from mtSSB^WT^ structure (PDB ID: 6RUP) was mutated *in silico* and, for both mtSSB^R107Q^ and mtSSB^WT^ structures, twenty models were automatically generated with the single mutant routine implemented in Modeller (14). The electrostatic interactions of arginine and glutamine were taken into account by setting the electrostatic restraints shell to 15 Å, to optimize the side chain orientation based in both stereochemical clashes and electrostatic contacts. To place the DNA from *Ec*SSB protein structure (PDB ID: 1EYG) onto the structure of the obtained in silico mutant mtSSB^R107Q^, we first superposed the *Ec*SSB dimer chains CD (with the longest ssDNA chain traced) onto mtSSB^R107Q^ AB dimer, with Coot (41). Once the two tetramers superposed, the *Ec*SSB was discarded and the DNA added to the mtSSB^R107Q^ coordinates. The structures were represented with Chimera (42), and the electrostatic potential surfaces were calculated with default parameters.

## Results

### The R107Q variant can form tetramers in solution and does not alter the ability of mtSSB to bind short ssDNA templates

**Figure 1.**
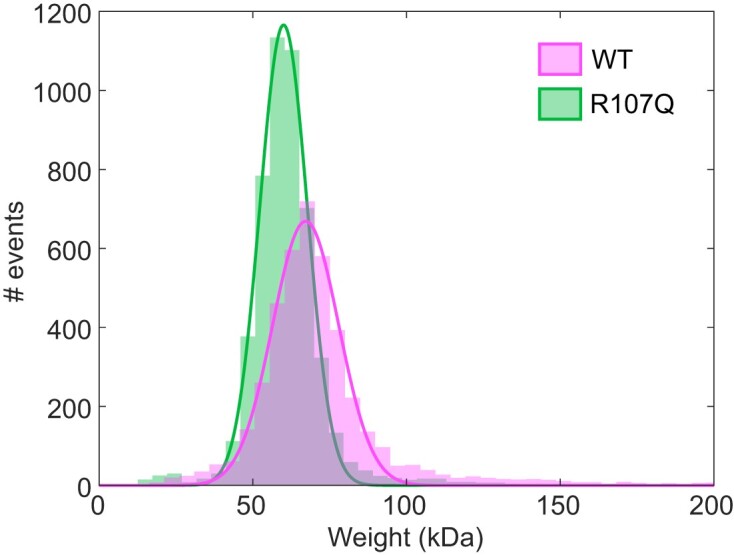
Mass photometry (MP) analysis of recombinant mtSSB^WT^ and mtSSB^R107Q^. Histograms showing the population distributions of mtSSB^WT^ (magenta) and mtSSB^R107Q^ (green) were fitted with a Gaussian, yielding a mean molecular weight of 67 ± 2 kDa for mtSSB^WT^ and 60 ± 2 kDa for mtSSB^R107Q^.

One molecular mechanism that could explain the pathogenicity of the mtSSB related diseases is a destabilization of the mtSSB tetramer into dimers or monomers. To test this hypothesis, we used mass photometry (MP) to assess the molecular mass and thus the oligomeric state of mtSSB^R107Q^ in solution (Figure 1). From fitting a Gaussian function to the obtained mass distribution, we found an absolute molecular mass of 67 ± 2 kDa for mtSSB^WT^ and 60 ± 2 kDa for mtSSB^R107Q^ (mean ± SD). Knowing that the molecular weight of one mtSSB tetramer containing the His-Tag is ∼65 kDa, these results indicate that the R107Q mutation does not impair the ability of mtSSB to form tetramers in solution.

Next, we set out to compare the ssDNA-binding affinity of mtSSB^WT^ and mtSSB^R107Q^ using biolayer interferometry and employing short ssDNAs of 30 and 60 nt respectively. We used sensors functionalized with either a 30-mer or 60-mer poly-dT and four different mtSSB concentrations (0.625, 1.5, 2.5 and 5 nM). In both cases, the binding traces of the four mtSSB concentrations were fitted with a 1:1 binding model (Supplementary Figure S2). We observed closely matched *K*_d_ values for both mtSSB^WT^ (*K*_d_ = 1.17 ± 0.02 nM) and mtSSB^R107Q^ (1.18 ± 0.02 nM) when interacting with a 60-mer poly-dT. Similarly, when a shorter 30-mer poly-dT oligonucleotide was used, the *K*_d_ values fell within a similar range, with *K*_d_ = 2.18 ± 0.10 nM for WT and *K*_d_ = 1.35 ± 0.04 nM for mtSSB^R107Q^. However, because these experiments were performed on short DNA templates, they did not provide information on the more complex dynamics of mtSSB-coated long ssDNA molecules that form *in vivo* during mtDNA transactions. We thus used single-molecule imaging techniques to further examine the DNA binding of mtSSB^WT^ and mtSSB^R107Q^.

### The R107Q mutation alters the ability of mtSSB to compact ssDNA

**Figure 2.**
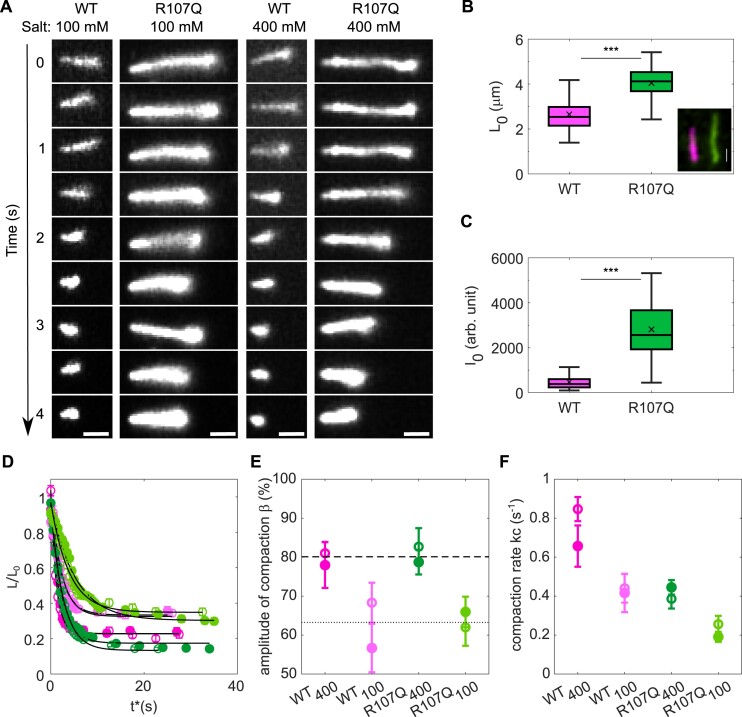
mtSSB^R107Q^ has reduced ssDNA compaction activity. (**A**) Selected frames from movies recording ssDNA compaction by mtSSB^WT-Alexa555^ or mtSSB^R107Q-Alexa555^ at two different salt concentrations (100 and 400 mM NaOAc) (scale bar, 1 μm). (**B**) Initial ssDNA length measurement at t0 before adding salt. Median values of L0 are 2.56 μm (*n* = 131) and 4.14 μm (*n* = 147) for WT and R107Q, respectively. Example image of a two-color experiment (inset) showing one ssDNA covered by mtSSB^WT-Alexa647^ (magenta) and one ssDNA molecule covered by mtSSB^R107Q-Alexa555^ (green) (scale bar, 1 μm). (**C**) Initial intensity of mtSSB-coated ssDNA at t0 before adding salt. Median values for WT and R107Q are 411 a.u. (*n* = 59) and 2745 a.u. (*n* = 59), respectively. In (**B**, **C**), boxplots represent the median within the IQR and *P*-values were calculated using unpaired Student's *t*-tests, ***P < 0.001. (**D**) For each condition, ssDNA length normalized by L0 averaged over 40 to 90 ssDNA molecules measured in three different experiments as a function of time showing an exponential decay (dark green: WT at 400 mM salt, light green: WT at 100 mM salt, dark pink: R107Q at 400 mM salt, light pink: R107Q at 100 mM salt). Open and closed symbols of the same color represent two different replicates. Error bars are the standard deviation. (**E**) Amplitude of compaction (β) in percentage of initial length for WT and R107Q at 100 and 400 mM salt. (**F**) Compaction rate (kc) for WT and R107Q at 100 and 400 mM salt. For (E) and (F), amplitude and compaction rates are obtained from an exponential fit to the data (black curves in (D)), and error bars indicate a 95% confidence level.

Knowing that tetramer destabilization and binding to short ssDNA templates are not the molecular mechanisms explaining the R107Q phenotype, we then investigated the interaction of mtSSB with longer, more physiological, ssDNA substrates. For this, we used an inverted fluorescence microscope in combination with a camera which allows imaging of a large field of view at the cost of single-molecule resolution (for details see (39)). We first visualized the effect of mtSSB on DNA compaction. To this end, we used 48.5 kb long biotinylated λ-phage DNA which has been converted into ssDNA using NaOH. The long ssDNA molecules were pre-incubated with fluorescence-labelled mtSSB^Alexa555^ (WT or R107Q, 150 nM) and formed nucleoprotein complexes were then tethered to the glass surface of a flow-cell on one end *via* a biotin-streptavidin bond and gently flow-stretched. The movies were started and, subsequently, salt (100 or 400 mM NaOAc) was added to the flow-cell and its effect on ssDNA compaction by mtSSB was monitored. The phenomenology was as follows: first the ssDNA was stretched by the flow to a nearly constant length, then, only a few seconds after the salt was added, we observed an important shortening of the ssDNA molecules, first rapidly and then slower; both for mtSSB^WT^ and mtSSB^R107Q^ (Figure 2A and Supplementary movie S1 and S2). For each experiment, we recorded a field of view with between 10 and 40 ssDNA molecules, which all decreased in length after addition of salt.

To quantify the ssDNA compaction observed in the movies, we determined, in each frame, the ‘apparent’ contour length (corresponding to the visible length of the DNA) and the total intensity of each ssDNA molecule. In total, we followed about ∼30 ssDNA molecules over eight independent experiments. The initial length (L0), obtained for each ssDNA by averaging its length in the first three frames of the movie, was L0 ∼2.56 μm for the WT-coated ssDNA, which was significantly shorter than the L0 ∼4.14 μm obtained for the ssDNA coated by mtSSB^R107Q^ (Figure 2B). As a control under the same experimental conditions in absence of mtSSB, we performed experiments with SYBR Green II DNA intercalator. Single-stranded DNA was pre-incubated with SYBR Green II for 20 min and added to the flow-cell in the same conditions as above, and the flow-cell washed to remove the free dye. The lengths of 115 flow-stretched SYBR-ssDNA molecules were measured, which yielded an average length of ∼8.5 μm (Supplementary Figure S3). Considering that the theoretical size of a 48.5 kb naked ssDNA is 16 μm, this implies that our flow-stretch protocol stretches the ssDNA to about half of its theoretical length. The initial (salt independent) compaction of the ssDNA by mtSSB^WT^ is thus about 70%, while for mtSSB^R107Q^ the initial ssDNA compaction is ∼50%. We also quantified the initial intensity (I0) of the nucleoprotein complexes in the first 3 frames. We observed that ssDNA-mtSSB^R107Q-Alexa555^ had significantly higher absolute intensities (2745 a.u.) than ssDNA-mtSSB^WT-Alexa555^ (411 a.u.), for the same illumination and acquisition settings (Figure 2C). This suggests that, at the same bulk concentration and in the absence of salt, ssDNA molecules are more covered by the mutant than by the WT.

We noted that rinsing with salt (100 and 400 mM) almost immediately removed the SYBR Green II from the ssDNA, it was thus not possible to follow the salt dependent compaction of the naked ssDNA. Therefore, we focused on the difference between the salt-dependent compaction by mtSSB^WT^ and mtSSB^R107Q^. For every ssDNA, we normalized its length in a frame L(t) by its initial length L0, obtaining the ratio L(t)/L0. The time at which the salt begins to have an effect t0* was determined as described in Supplementary Figure S4. Next, L(t*)/L0 was averaged over all ssDNA molecules and represented in Figure 2D as a function of t*, where t*=t-t0*. To describe the experimental data we fitted the exponential decay function LL0=β ×exp-kc × t*+LfL0, where β, kc are the amplitude and rate of the salt dependent compaction, respectively, and Lf the average final length of the compacted ssDNA (Figure 2D). The amplitudes of ssDNA compaction β are similar for mtSSB^WT^ and mtSSB^R107Q^, and both appear only dependent on the salt concentration, increasing from about 63% at lower salt (100 mM) to 80% at high salt (400 mM) (Figure 2E). In contrast, the rates of compaction kc depend both on the salt concentration and on the mtSSB used, with an almost twice faster compaction rate for mtSSB^WT^ than for mtSSB^R107Q^ (Figure 2F). Additionally, for both mtSSB^WT^ and mtSSB^R107Q^, at high salt concentration the compaction is twice faster than at low salt. This suggests that ssDNA compaction by mtSSB is salt-dependent and is more efficient for mtSSB^WT^ than for mtSSB^R107Q^.

Finally, to rule out the possibility that the nucleoprotein complexes were differently stretched in the different experiments, we also performed a two-color experiment, with ssDNA-mtSSB^WT-Alexa647^ and ssDNA-mtSSB^R107Q-Alexa555^ flushed and stretched simultaneously on the same glass (Figure 2B, inset). Here again, we confirmed a higher compaction of ssDNA with mtSSB^WT^ than with the mutant.

**Figure 3.**
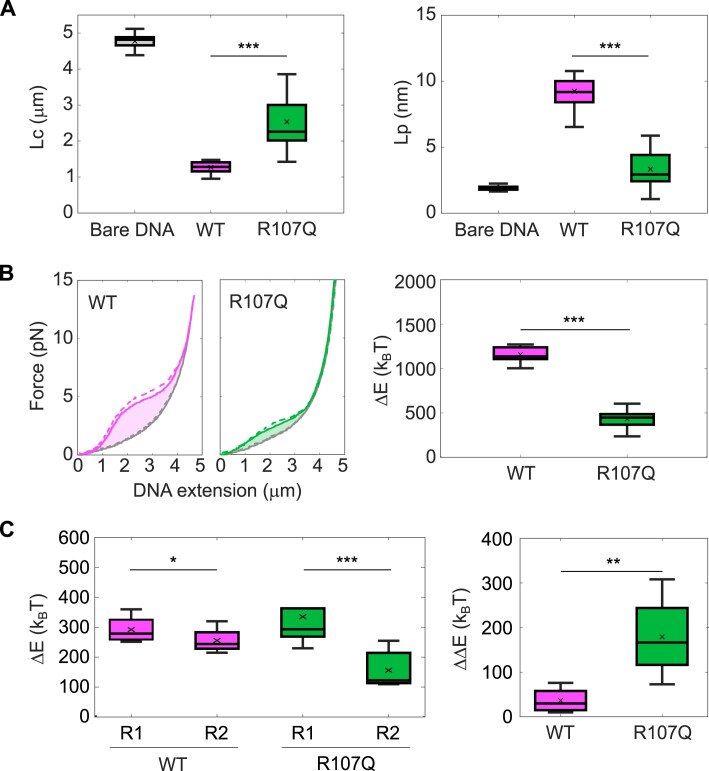
The R107Q mutation alters the ssDNA-binding properties of mtSSB. (**A**) Estimation of the contour length (Lc) and the persistence length (Lp) of bare ssDNA and ssDNA incubated with 100 nM of mtSSB^WT^ or mtSSB^R107Q^, using the eFJC model on the corresponding force-distance retraction curves obtained with AFS. Fitting range for bare DNA curve: 0.1–15 pN and for mtSSB-coated DNA: 0.1–2 pN. (**B**) Force-distance extension (dashed lines) and retraction (solid lines) curves of ssDNA in presence of mtSSB^WT^ (magenta) and mtSSB^R107Q^ (green), compared to bare DNA (dark grey). The area between the retraction curve obtained in the presence of mtSSB and the retraction curve obtained for bare ssDNA was used to calculate the change in energy (Δ*E*) due to mtSSB-binding to ssDNA. (**C**) The area between the force–distance extension and retraction curves obtained in the presence of mtSSB when stretching the DNA with two successive ramps R1 and R2 (see Supplementary Figure S5C) at low force (6–7 pN) was used to calculate the Δ*E* due to mtSSB–ssDNA interaction at *R*1 and *R*2, and the ΔΔ*E*, which represents the difference between Δ*E* at *R*1 and Δ*E* at *R*2. Boxplots represent the median within the IQR for (A, B) a minimum of 20 tethers acquired in three different experiments for each condition or (**C**) a minimum of eight tethers acquired in two different experiments for each condition. *P*-values were calculated using (A, B) unpaired Student's *t*-tests and (C) paired Student's *t*-tests, where *P < 0.05, **P < 0.01, ***P < 0.001. The Δ*E* has been converted from pN•nm to k_B_T (i.e. the Boltzmann constant multiplied by the absolute temperature) using the relationship k_B_T ∼4.1 pN•nm at 25°C (44).

Having shown that mtSSB^R107Q^ is less efficient in compacting ssDNA, we set out to quantify in more detail its ability to bind ssDNA, using acoustic force spectroscopy (AFS). For these experiments, we used 9-kb long ssDNA molecules attached on one end to the glass surface and on the other end to a bead. We performed force-distance extension (DNA stretching with an increasing force ramp) and retraction (DNA relaxation with a decreasing force ramp) experiments on bare ssDNA and on mtSSB-coated ssDNA. Each force ramp was repeated twice, which limits unspecific interactions of the ssDNA molecules with the flow-cell surface at the beginning of the extension phase for the second ramp (Supplementary Figure S5A). We observed that the force–distance curves for mtSSB-coated ssDNA significantly deviated from the curves corresponding to bare DNA, with a clearly stronger effect for the WT than for the mutant (Representative DNA molecules shown in Supplementary Figure S5B). We quantified the effect of mtSSB-binding on the mechanical properties of the ssDNA by fitting the force-distance retraction curves with the extensible Freely Jointed Chain model (eFJC), commonly used to model the relation between applied tensions and ssDNA extension (40). For bare ssDNA, we fitted the force-distance curves from 0.1 to 15 pN and obtained values of apparent contour length Lc of 4.8 μm ± 0.2 and persistence length Lp of 1.9 nm +/- 0.3 (mean +/- SD). To estimate the Lc and Lp values after addition of mtSSB, we fitted only the first part of the force-distance curves, which deviated from the curve with bare ssDNA, from 0.1 to 2 pN, as done previously by Xu *et al.* (23). In the presence of mtSSB^WT^, we observed that the Lc decreased to 1.3 μm ± 0.2 while the Lp increased to 9.3 nm ± 1.8. In the presence of mtSSB^R107Q^, the decrease in Lc was less drastic than in the presence of mtSSB^WT^ (Lc = 2.5 μm ± 0.8) and the Lp was only slightly higher than the Lp for bare DNA (Lp = 3.3 nm ± 1.2) (Figure 3A and Supplementary Figure S5B). The Lc value for mtSSB^WT^-coated ssDNA is thus ∼4 times smaller than the Lc of bare DNA, while with mtSSB^R107Q^ the Lc is only ∼twice smaller, which confirms that R107Q alters the compaction capacity of mtSSB. Meanwhile, the Lp values, which represent the bending stiffness of the ssDNA, indicate that ssDNA covered by mtSSB^WT^ is ∼5 times stiffer than bare ssDNA, while ssDNA covered by mtSSB^R107Q^ has a similar rigidity than bare ssDNA. This AFS experiment can also be used to determine the energetic consequences of protein binding to DNA (43), with the area under the force-distance curve being the work performed on the DNA that is stretched (30). We therefore integrated the area between the retraction curve obtained in the presence of mtSSB and the retraction curve obtained for bare ssDNA, to calculate the change in energy due to mtSSB-binding to ssDNA. The work stored in the mtSSB^R107Q^-coated ssDNA was almost 3 times smaller than in the mtSSB^WT^-coated ssDNA, indicating an alteration of the mtSSB-binding capacities (Figure 3B). Finally, we investigated the force-dependency of mtSSB-binding to ssDNA. To this aim, we applied lower amplitude force-ramps after addition of mtSSB in the flow-cell (Supplementary Figure S5C). In this configuration, the stretching and relaxing force-distance curves overlapped for tensions below 3–4 pN (ramps R’1 and R’2 in Supplementary Figure S5C), while we observed hysteresis (defined as a difference in the force-distance extension and retraction curves of the same force ramp) for higher tensions of 6–7 pN (ramps R1 and R2), for both WT and mutant proteins. Although hysteresis was observed for both WT and R107Q proteins for forces of 6–7 pN, the mtSSB^R107Q^ appeared less stable on the ssDNA, as the force distance-curves obtained with two consecutive ramps at this tension did not overlap (ramps R1 and R2, Supplementary Figure S5C), indicating that some mtSSB^R107Q^ molecules detached from the DNA. This was quantified by integrating the area between the force-distance extension and retraction curves obtained with the two successive ramps R1 and R2, and subtracting the integrated area for R1 and the integrated area for R2 (change in energy ΔE and ΔΔE, respectively, Figure 3C), which confirmed that the change in energy due to mtSSB–ssDNA interaction was significantly lower for mtSSB^R107Q^ during the second ramp R2 compared to the first ramp R1. The force experiments thus indicate that the R107Q mutation significantly reduces mtSSB ability to compact ssDNA, by altering mtSSB–ssDNA interactions and increasing its dissociation at mild tensions.

### Disassembly of mtSSB^R107Q^ from ssDNA

**Figure 4.**
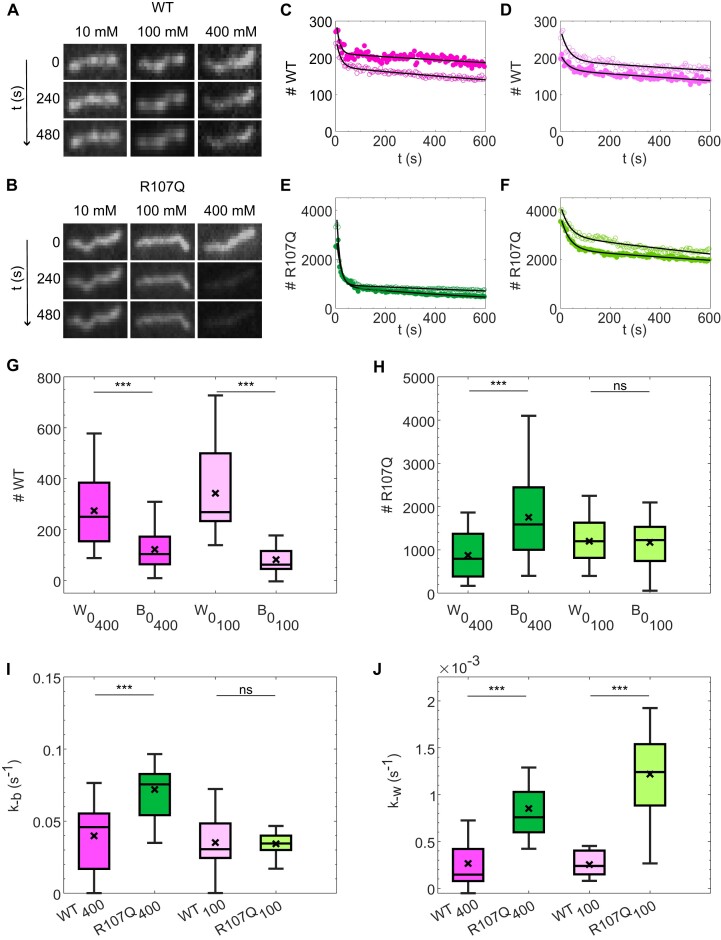
Disassembly of mtSSB from ssDNA is faster for mtSSB^R107Q^ than for mtSSB^WT^. Typical fluorescence images of stretched mtSSB^WT^ (**A**) or mtSSB^R107Q^ (**B**) -ssDNA complexes at different NaOAc concentrations (10, 100 and 400 mM) at indicated time intervals. Images were acquired with an exposure time of 0.1 s every 5 s to limit the effects of photobleaching. Fluorescence intensity was converted into number of mtSSB monomers as described in Supplementary Figures S7 and S8 and the number of mtSSB monomers bound was followed over time at different salt concentrations (C–F). Example traces for mtSSB^WT^ at 400 mM NaOAc (**C**), mtSSB^WT^ at 100 mM NaOAc (**D**), mtSSB^R107Q^ at 400 mM NaOAc (**E**), mtSSB^R107Q^ at 100 mM NaOAc (**F**). Open and closed symbols of the same color represent two different replicates, black curve: fit to the data. All traces are shown in Supplementary Figure S9. Initial number of molecules in the wrapped state w0 and in the bound state b0, in experiments where 100 or 400 mM NaOAc was subsequently added, for mtSSB^WT^ (**G**) and mtSSB^R107Q^ (**H**). Unbinding rate k-b (**I**) and unwrapping rate k-w (**J**). (G–J) The box plots represent the median with the IQR of the parameters obtained by fitting 30–60 ssDNA molecules acquired in three different experiments for each condition. Both mtSSB^R107Q^ and mtSSB^WT^ were labelled with Alexa 555.

During DNA compaction by the proteins, we noticed a decrease in the fluorescence intensity over time of mtSSB^R107Q^-coated ssDNA molecules, especially in presence of high salt concentration, whereas the intensity of mtSSB^WT^-coated ssDNA remained stable (Supplementary Figure S6). An enhanced dissociation of mtSSB^R107Q^ from ssDNA was also observed in the force experiments (Figure 3C). Together, these results could suggest that, in the presence of salt, a fraction of the R107Q protein dissociates from ssDNA, and led us to investigate, in detail, the salt-dependent disassembly kinetics of mtSSB from ssDNA. Pre-formed nucleoprotein complexes were flushed in a flow-cell, tethered to the glass surface on one end via a biotin-streptavidin bond and, using a high flow rate, attached to the glass surface through nonspecific interactions. This resulted in most of the ssDNA molecules being fixed and only a small fraction remaining loose or breaking. We then followed in real-time for three different salt concentrations (10, 100 and 400 mM) the decreasing intensity of the mtSSB-coated-ssDNA molecules, reflecting the dissociation from ssDNA of mtSSB^WT-Alexa555^ and mtSSB^R107Q-Alexa555^ (see representative images in Figure 4A and B). To limit the effect of photobleaching, we acquired fluorescence images intermittently every 5 s. For each image, we converted the fluorescence intensity into the number of mtSSB molecules on a ssDNA molecule by summing the fluorescence intensity on the DNA and dividing it by the intensity of a single fluorophore (see Supplementary Figures S7 and S8).

The time evolution of the number of mtSSB^WT^ and mtSSB^R107Q^ molecules on ssDNA displayed two regimes: first (i) a fast decrease, followed by (ii) a slower decrease (Figure 4C–F and Supplementary Figure S9). Since the fluorescence of the mtSSB-coated-ssDNA remained nearly constant after adding 10 mM salt (Supplementary Figure S9), we focused our analysis on the salt concentrations of 100 and 400 mM. We estimated that, for mtSSB^WT^, the number of molecules on ssDNA decreased over time by about 20%, independently of the salt concentration. Instead, for mtSSB^R107Q^, this loss was ∼40% at 100 mM salt and ∼77% at the highest salt concentration (Figure 4D and F). The sharp transition between the two regimes could not be captured by a simple one-state transition which would result in a single exponential function. Therefore, we described our data using a two-state model with a high binding mode (W, wrapping mode) and a low binding mode (B, binding mode) and assumed that dissociation happens only from the low binding mode described above (15,16) in the sequence W→B→0 (0 being no binding). For each ssDNA, the number of mtSSB molecules was fitted using the following Ordinary Differential Equations (ODE)s: dwdt = k-ww and dbdt = k-ww-k-bb, where w and b correspond to the number of molecules in the wrapping and binding state, respectively, and k-b and k-w, the unbinding and unwrapping rates, respectively. We found that this simple two-state model described our data well (fit in black Figure 4C–F). The obtained fitting parameters w0 and b0, corresponding to the initial number of molecules in the wrapped and bound state, i.e. before the added salt starts to have effect, and the rates k-w and k-b are depicted in Figure 4G–J. The unbinding and unwrapping rates were corrected for photobleaching by subtracting the significantly smaller kbleaching ∼5.9e-3 s^−1^ (see Supplementary Figure S8). Overall, the total number of mtSSB molecules on a ssDNA before addition of salt was larger for mtSSB^R107Q^ (∼2200 molecules) than for mtSSB^WT^ (∼500 molecules), consistent with what we observed in our compaction experiments. For mtSSB^R107Q^, the number of molecules in the binding mode was similar to those in the wrapping mode (b0∼w0∼1100 molecules), while for mtSSB^WT^ there were twice more molecules in the wrapping mode than in the binding mode (w0∼2*b0∼300 molecules) for both 100 mM and 400 mM salt (Figure 4G, H). The unbinding rates k-b were similar for mtSSB^WT^ at 100 mM and 400 mM salt, and mtSSB^R107Q^ at 100 mM salt, however it was increased for the mtSSB^R107Q^ at high salt. As for the unwrapping rates k-w, they appeared not to depend on the salt, but were almost three times slower for the mtSSB^WT^ than for the mtSSB^R107Q^. In conclusion, we observed that in general the dissociation of the mtSSB from ssDNA shows two regimes, and that mtSSB^R107Q^ dissociates faster from ssDNA than mtSSB^WT^.

### Competition assays

**Figure 5.**
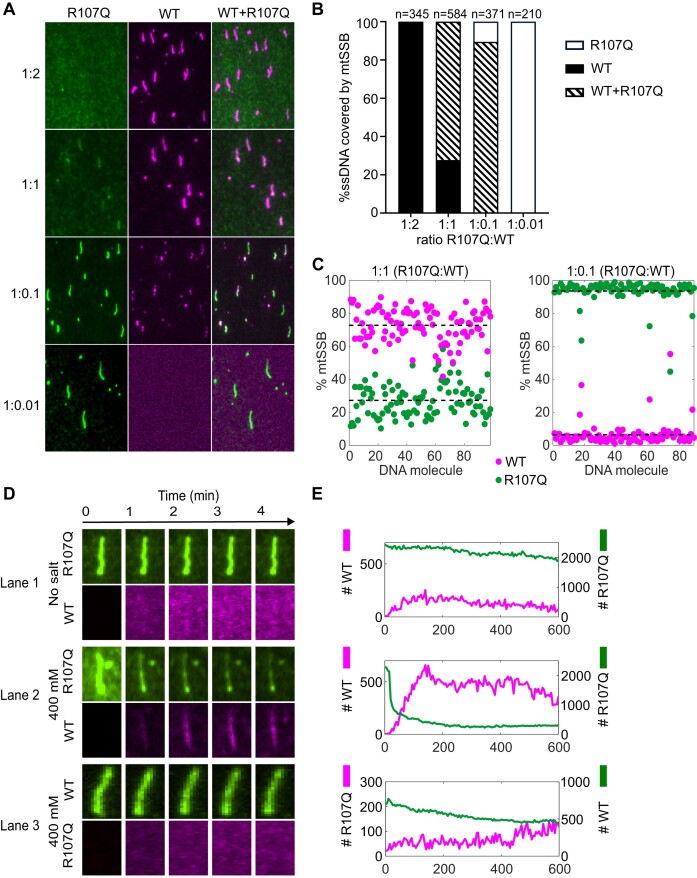
Binding competition between mtSSB^WT^ and mtSSB^R107Q^. (A–C) Binding competition at different mtSSB^R107Q-Alexa555(green)^:mtSSB^WT-Alexa647(magenta)^ protein concentration ratios. (**A**) Representative images of nucleoprotein complexes at different concentration ratios: 1:2, 1:1, 1:0.1, 1:0.01 (R107Q:WT). The mix of the two proteins was incubated with ssDNA for 60 min and the nucleoprotein complexes formed were attached on the glass surface. Image contrasts were adjusted differently for each ratio to visualize low abundance proteins. (**B**) Histogram showing in percentage the number of ssDNA molecules covered by only mtSSB^WT^, both mtSSB^WT^ and mtSSB^R107Q^ or only mtSSB^R107Q^ in each condition. (**C**) Quantification of mtSSB molecules on ssDNA molecules coated by both mtSSB^WT^ and mtSSB^R107Q^, at 1:1 (left) and 1:0.1 (right) ratios. (D, E) Real-time competition between mtSSB^R107Q^ and mtSSB^WT^. (**D**) TIRF images of nucleoprotein complexes over time in three different settings. The upper line of each configuration (1–3) shows mtSSB^R107Q-Alexa555^ or mtSSB^WT-Alexa555^–ssDNA complexes stretched at the surface of the flow-cell. The lower line shows mtSSB^R107Q-Alexa647^ or mtSSB^WT-Alexa647^ that is added to the flow-cell in the presence of salt. (**E**) Intensity measurement of mean intensities of ssDNA–mtSSB complexes as a function of time for each configuration (1–3).

In patients, R107Q is a dominant, heterozygous variant, which implies that the mutant protein coexists with the WT version of mtSSB. We thus developed a dual color competition assay between mtSSB^WT^ and mtSSB^R107Q^ for ssDNA binding. In this assay, mtSSB^R107Q-Alexa555^ (150 nM) was mixed with increasing concentrations of mtSSB^WT-Alexa647^ (1.5, 15, 150 and 300 nM; corresponding respectively to the concentration ratios 1:0.01, 1:0.1, 1:1 and 1:2). Figure 5A shows, for each experimental condition, typical two-color TIRF images as well as an overlay of those images. Images were analyzed as follows: for each protein ratio, we counted the DNA molecules covered with only mtSSB^WT^ (Figure 5B, black), both mtSSB^WT^ and mtSSB^R107Q^ (Figure 5B, stripes), or only mtSSB^R107Q^ (Figure 5B, white) and expressed them as a percentage of the total number of counted DNA molecules. In total, we counted between 210 and 345 DNA molecules for each condition. We found that, when the mtSSB^WT^ concentration is higher than the mtSSB^R107Q^ concentration (1:2 ratio, R107Q:WT), only mtSSB^WT^ was bound to ssDNA (Figure 5B). However, at a 1:1 ratio, we noticed that 24% of the ssDNA molecules were covered exclusively by mtSSB^WT^ whereas 76% of the DNA molecules were covered by both the WT and the mutant proteins (Figure 5B). At this ratio, we could not detect any ssDNA molecules coated exclusively with mtSSB^R107Q^. Only when a 10-fold excess of mtSSB^R107Q^ was added, some ssDNA molecules exclusively coated by mtSSB^R107Q^ could be observed (∼10%) and 90% of the ssDNA was coated by both proteins. Finally, with a 100-fold excess of mtSSB^R107Q^ (1:0.01 ratio), no mtSSB^WT^ could be detected on the DNA. We then focused on the ratio 1:1 and 1:0.1 and took a closer look at the ssDNA molecules coated by both mtSSB^WT^ and mtSSB^R107Q^. We determined the number of mtSSB^WT^ and mtSSB^R107Q^ on each DNA molecule from the total fluorescence intensity on the DNA and the intensity of a single fluorophore (Figure 5C). For the 1:1 ratio, we noticed that the co-coated ssDNA molecules are in fact mostly coated by the WT (73%) (Figure 5C), showing that at this ratio there is mainly mtSSB^WT^ bound to DNA. For the 1:0.1 ratio, the co-coated ssDNA molecules are instead mostly covered by mtSSB^R107Q^ (94%).

Next, we set out to follow the competition between the two proteins in real-time. In this experiment, we attached preformed mtSSB^R107Q-Alexa555^–ssDNA or mtSSB^WT-Alexa555^–ssDNA nucleoprotein complexes at the surface of the flow-cell, then added mtSSB^WT-Alexa647^ or mtSSB^R107Q-Alexa647^, respectively, in the presence of different salt concentrations (0 or 400 mM) and followed the proteins on the DNA over 10 min (Figure 5D, E). In the absence of salt, mtSSB^WT^ binds only slightly to mtSSB^R107Q^-coated ssDNA molecules (Figure 5D, lane 1). In contrast, in presence of 400 mM NaOAc, mtSSB^R107Q-Alexa555^ dissociates from ssDNA and free WT proteins (magenta) bind rapidly on ssDNA (Figure 5D, lane 2 and Supplementary movie S3). When performing the opposite experiment, i.e. competing pre-formed mtSSB^WT-Alexa555^–ssDNA complexes with free mtSSB^R107Q-Alexa647^ (Figure 5D lane 3 and Supplementary movie S4), we observed only a slight dissociation of WT and almost no binding of mtSSB^R107Q^. When we injected mtSSB^R107Q-Alexa647^ in presence of 400 mM NaOAc on pre-formed ssDNA–mtSSB^R107Q-Alexa555^ complexes, we noticed that, even if mtSSB^R107Q-Alexa555^ dissociates from ssDNA, only a relatively small fraction of the ssDNA is coated again by mtSSB^R107Q-Alexa647^ (Supplementary Figure S10 lane 1). Finally, when we injected mtSSB^WT^ in presence of 400 mM NaOAc on pre-formed ssDNA–mtSSB^WT^ complexes, some WT protein can bind even though there is nearly no dissociation, indicating that under these experimental conditions there is still some free space on the ssDNA (Supplementary Figure S10, lane 2). Competition assays thus show that mtSSB^WT^ dominates ssDNA binding over mtSSB^R107Q^.

### Modeling of R107Q mutation and potential DNA contacts on mtSSB structure

**Figure 6.**
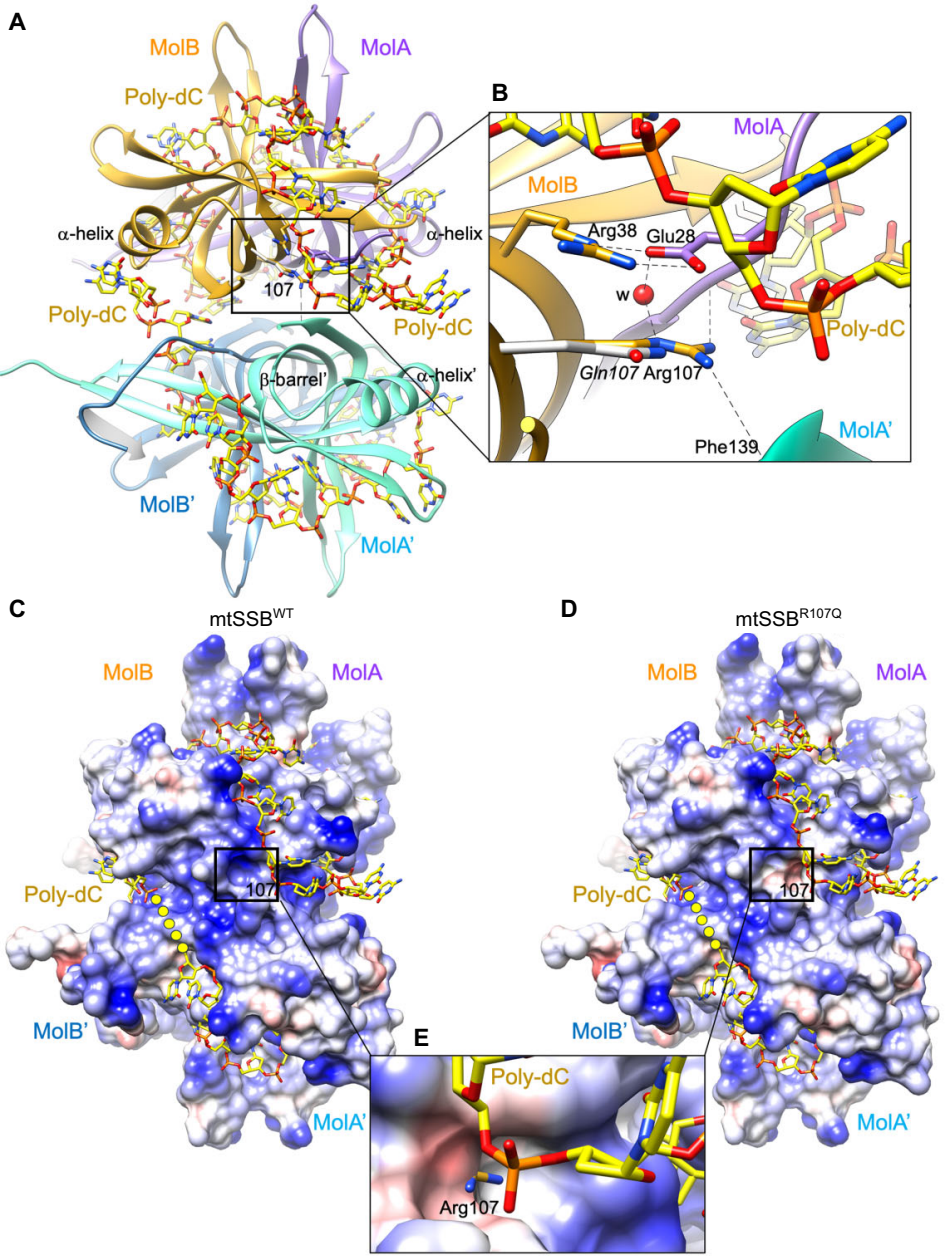
Analysis of the effects of the R107Q mutation on the structure of mtSSB. (**A**) Two molecules MolA (colored in violet) and MolB (in gold) are related by a two-fold axis, forming a dimer that is related by crystallographic symmetry to a second dimer (MolB’ and MolA’, in light and dark blue, respectively), generating the physiological tetramer. By superposing the SSB protein from *E. coli* bound to a poly-dC 35-mer (PDB ID: 1EYG) onto the mtSSB structure (6RUP), the ssDNA fragments originally traced in *Ec*SSB locate on the mitochondrial mtSSB tetramer, covering the region of Arg107 residue (framed). The β-barrel is indicated for MolA’, and so is the α-helix for monomer MolA, MolB and MolA’. (**B**) Close-up view showing the interaction of Arg107 with surrounding residues, which include Arg38 from the same molecule (by van der Waals interactions between the methylene groups of respective side chains), Glu27 from MolA, and Phe139 amide from MolA’. A water molecule (‘w’ red sphere) stabilizes the interactions. The modeled R107Q mutation (14) shows that the Gln shorter side chain changes the charge of the surface, from a positive charge to a polar side chain. Side chains of residues are represented in the color of the own molecule, the nitrogen and oxygen atoms in blue and red, respectively. (**C**, **D**) The electrostatic potential represented on a Conolly surface (electronegative and positive potentials shown in red and blue, respectively) of the mtSSB^WT^ and modeled mtSSB^R107Q^ structures, respectively. The surface at position 107 is framed, and the connection between DNA fragments is represented as yellow dots. Note that the positive electrostatic potential in the WT changes to negative potential in the mutant, near the ssDNA. (**E**) Close-up view of the R107Q electrostatic surface superposed to the WT structure. The surface around shorter Gln107 is electronegative and extends below the positively charged side chain of Arg107, whose charged tip protrudes.

The crystal structures of mtSSB (13,14,45) show a tetramer as a dimer of dimers (dimer molA/molB, and dimer molA’/molB’, respectively) (Figure 6A). Each monomer is constituted by a β-barrel whose β-strands are connected by long β-hairpins and an α-helix (OB-fold). In the dimer, the two β-barrels are connected and form a long hydrophobic tunnel capped at either end by the α-helix of the corresponding monomer. At one side of the two connected β-barrels the strands form a continuous β-sheet that contacts the same β-sheet of the other dimer in the tetramer. The overall arrangement shows one pair of β-hairpins protruding from each dimer, whereas the other β-hairpins are on the plane of the two-contacting β-sheets. Arg107 is precisely located at one edge of the flat β-sheet, at the interface between monomers in a dimer, and between the two dimers. In detail, Arg107 contacts molB from the same dimer by its guanidinium group: Arg107 Nϵ atom interacts with Glu27 carboxyl O2 whereas Arg107 NH2 contacts main chain carbonyl C=O of Arg28, both residues from molB (Figure 6B). A water molecule further stabilizes these contacts. At the second dimer, the contact occurs between Arg107 guanidinium group NH1 atom and the main chain C=O from molA’ Phe139. In all protomers, the methylene groups of Arg107 side chain make van der Waals interactions with Arg38 from the same molecule, the guanidinium group of this latter further stabilized by carboxyl Oϵ2 from aforementioned Glu27 from the other monomer. Remarkably, similar to Arg107, mutation at Arg38 is also associated with optic atrophy and foveopathy (14).

The structure of human mtSSB in complex with DNA is not available, therefore in order to get a hint on the regions of mtSSB potentially contacting DNA, we superposed onto human mtSSB (PDB ID: 6RUP) the crystal structure of *Ec*SSB tetramer (PDB ID: 1EYG), which is the SSB protein showing the highest sequence similarity with human mtSSB among deposited PDB structures (32% identity, 60% similarity for 111 amino acids aligned by BLAST). In this crystal structure, *Ec*SSB tetramer is bound to two poly-dC 35-mer, one per dimer (46), which, to the best of our knowledge, is the largest ssDNA fragment bound to an SSB protein with OB fold (Figure 6A). The two structures were superposed by their respective dimers AB (mtSSB) and CD (*Ec*SSB, with longest ssDNA traced), which automatically resulted in the superposition of the DNA from *Ec*SSB onto the mtSSB structure. Remarkably, the 35-mer structure, which is negatively charged due to the phosphate backbone, superposes onto a long electropositive patch from mtSSB that includes Arg38 and Arg107 residues (Figure 6C). The spatial coincidence of opposing charges between the *Ec*SSB DNA and mtSSB substantiates a similar interaction of ssDNA with both human and *E. coli* SSB molecules. In addition, the change from Arg107 to Gln results in an electronegative spot that interrupts the electropositive patch, reducing potential interaction sites (Figure 6C–E). The structural modeling of ssDNA bound to mtSSB thus suggests that the R107Q mutation directly impacts the stability of the protein–ssDNA interaction.

## Discussion

The R107Q mutation has been identified as a pathogenic mutation in 2019 in three different studies (14,32,33). The clinical features of patients carrying the R107Q mutation include variable combination of optic atrophy with clinical phenotypes including retinal macular dystrophy, sensorineural deafness and nephropathy (14,32,33). In muscle and kidney biopsies of affected patients carrying the R107Q mutation, a partial depletion of mtDNA was observed. Moreover, experiments performed on mitochondria isolated from primary fibroblasts of those patients showed a depletion of mtDNA, a lower amount of 7S DNA, and a slower DNA repopulation after ethidium bromide treatment (32). Electrophoretic mobility shift assay (EMSA) experiments also showed later that mtSSB^R107Q^ displayed decreased binding to a short 30-nt ssDNA fragment but a binding pattern similar to that seen with mtSSB^WT^ on a 100-nt ssDNA fragment, although mtSSB^R107Q^ had significantly higher off rate from ssDNA than mtSSB^WT^ (8). Here we have determined *K*_d_ values on 30-nt and 60-nt templates using BLI and found very similar *K*_d_ for mtSSB^WT^ and mtSSB^R107Q^ (*K*_d30-nt_ = 1.17 and 1.18 nM and *K*_d60-nt_ = 2.2 and 1.4 nM for mtSSB^WT^ and mtSSB^R107Q^, respectively). These values are very similar to the values found for mtSSB^WT^ using a 40-nt ssDNA and either fluorescence polarization methods (*K*_d_ = 1.8 ± 0.9 nM) (47) or EMSA (*K*_d_ = 2.3 nM) (48). However, studies on short templates are limited to the interactions on DNA of one or a few proteins. We thus moved to longer, more biologically relevant DNA lengths to better understand mtSSB binding dynamics.

It was shown that, depending on the mtSSB and salt concentrations, there are two binding modes for mtSSB to ssDNA, namely a wrapping mode and a binding mode (15,16,17,18). These modes have their own rates and footprints: the footprint in the binding mode is 30 bp while the footprint in the wrapping mode is 60 bp (15). Our dissociation experiments (Figure 4) revealed that mtSSB unbinding to ssDNA can indeed be modeled by a generic two-state binding model mtSSB.ssDNA(wrapped) → mtSSB.ssDNA(bound) → mtSSB + ssDNA. The wrapping and binding states have their own rate constants, and the hypothesis is that dissociation of mtSSB from ssDNA occurs only from the bound state. The addition of salt modulates the transition of mtSSB from the wrapped state to the bound state from which the mtSSB may dissociate from the ssDNA. Using this simple model, we found that there are initially an average of about 350 mtSSB^WT^ molecules bound per ssDNA molecule, of which 26% are in the binding mode and 74% in the wrapping mode (corresponding to 25 and 70 tetramers, respectively). Taking the footprint of a mtSSB tetramer in the binding mode of 30 bp and in the wrapping mode of 60 bp (15), this suggests that only about 10% of the 48.5 kb ssDNA is coated by the mtSSB^WT^. Instead, we found that around 2200 mtSSB^R107Q^ molecules are on the ssDNA in the dissociation experiments, and half of these are in the binding state while the other half is in the wrapping state. Therefore, there are about 275 mtSSB^R107Q^ tetramers in the wrapping state, while for mtSSB^WT^ there are only about 70 tetramers in the wrapping state, and yet the compaction of the ssDNA is more pronounced for mtSSB^WT^. This could suggest that the apparent footprint of mtSSB^R107Q^ is smaller than the footprint of mtSSB^WT^. Instead, we suggest that this lower apparent footprint of mtSSB^R107Q^ is likely related to a more transient wrapping state, as indicated by the 3-fold larger unwrapping rate (k-w) calculated for mtSSB^R107Q^ and by the force experiments, as we will discuss later. We therefore considered the same footprints for mtSSB^R107Q^ and mtSSB^WT^, which yielded a coverage of about 50% of the ssDNA for mtSSB^R107Q^. This higher coverage of the ssDNA was also qualitatively observed in the compaction experiments (Figure 2) where the ssDNA-mtSSB^R107Q^ complexes had a higher intensity than those with mtSSB^WT^.

A reduced ssDNA wrapping efficiency of mtSSB^R107Q^ was also evidenced in the compaction assays, where, for a given salt concentration, we found that the wrapping rate (kc) is almost a factor 2 higher for mtSSB^WT^ than for mtSSB^R107Q^. Therefore, it appears that the mutation R107Q mainly affects the wrapping state, resulting in a less efficient and less stable wrapping of ssDNA by mtSSB^R107Q^ than by mtSSB^WT^.

Our competition assays (Figure 5) showed that at a 1:1 ratio of mtSSB^WT^: mtSSB^R107Q^, about 76% of the ssDNA is co-covered by mtSSB^WT^ and mtSSB^R107Q^ and about 24% is exclusively covered by mtSSB^WT^. At this ratio, 73% of the mtSSB on the co-covered ssDNA is mtSSB^WT^ and 27% is mtSSB^R107Q^. If we extrapolate to an *in vivo* situation, this will imply that for a heterozygous patient, ssDNA should be predominantly interacting with mtSSB^WT^. Only when there is a 10-fold excess of mtSSB^R107Q^ most of the ssDNA is co-covered with 90% mtSSB^R107Q^ and 10% mtSSB^WT^. There should thus exist a sharp threshold above which mtSSB^R107Q^ becomes dominant. However, it is also possible that, *in vivo*, heterotetramers containing WT and mutated subunits are formed, leading to the dominant negative effect observed in patients. We also showed that for a ssDNA covered by mtSSB^R107Q^ in the absence of salt, mtSSB^R107Q^ does not readily dissociate from the ssDNA and mtSSB^WT^ appears not to be able to bind. This would suggest that the ssDNA is completely covered by mtSSB^R107Q^. However, the total number of mtSSB^R107Q^ molecules (∼2200) corresponds to a ssDNA coverage of about 50%, which implies that there should be 50% of free ssDNA for mtSSB^WT^ to bind to. The observation that no mtSSB^WT^ can bind may indicate that the free ssDNA is divided into small stretches between neighboring mtSSB^R107Q^ molecules and that those stretches are too small for the footprint of mtSSB^WT^ (∼60 bp). In the presence of salt, mtSSB^R107Q^ dissociates rapidly from the ssDNA, making space for mtSSB^WT^ to attach to ssDNA. On the contrary, ssDNA covered with mtSSB^WT^ is more stable, and the addition of salt does not induce the dissociation of the mtSSB^WT^. Even though these nucleoprotein complexes are not saturated, the mtSSB^R107Q^ cannot stably bind under the high salt conditions.

To interpret our force spectroscopy data and infer the mechanical properties of ssDNA ± mtSSB we used the eFJC model. This model can be applied to our data, as the DNA remains flexible between bound mtSSB molecules (49), and allowed us to retrieve the changes in contour length Lc and persistence length Lp after adding mtSSB to bare ssDNA (Figure 3). For the Lc and Lp estimations, we used the force–retraction curves rather than the force-extension curves, as ssDNA can bind non-specifically to the flow-cell at very low forces. We did not observe the formation of noticeable ssDNA secondary structures, which would be indicated by hysteresis between the force-extension and the force-retraction curves of the bare ssDNA, as reported before (30). Binding of mtSSB^WT^ to ssDNA significantly reduced the Lc at low forces, indicating shortening of the DNA molecules through compaction, probably involving the wrapping mode of mtSSB. These results are in line with Kaur *et al.*, where a shortening of the Lc of DNA was observed in presence of mtSSB, that was attributed to DNA wrapping around human mtSSB (47). This reduction was less striking after addition of mtSSB^R107Q^, confirming the alteration of DNA compaction ability, also seen with our single-molecule fluorescence microscopy. For the persistence length Lp, we obtained a value of 1.9 nm for bare ssDNA in presence of 100 mM salt, which is consistent with the literature (1.3 nm for Tinland *et al.* (50) and 2.5 nm for Murphy et *al*. (51), at the same salt concentration). mtSSB^WT^-binding increased the Lp ∼5 times, to 9.3 nm, indicating that protein-binding strongly decreases the flexibility of the ssDNA. However, the Lp value after addition of mtSSB^R107Q^ is only slightly increased compared to the Lp value of bare ssDNA, to 3.3 nm. The persistence length is directly linked to the Kuhn length, which corresponds to the length of the inflexible segment of the polymer chain. In the case of bare ssDNA, this inflexible subunit is the nucleotide, while after binding of mtSSB, it is the footprint of the protein. The significantly lower Lp value for mtSSB^R107Q^ compared to mtSSB^WT^ indicates a smaller apparent footprint of the mutated protein, which can be explained by a less stable wrapping state for mtSSB^R107Q^, as we discussed previously.

Additionally, we investigated the force-dependent stability of the mtSSB–ssDNA complexes. The force at which mtSSB starts to detach can be deducted from the force–distance curves, by applying cycles of stretch–relax with increasing forces to the ssDNA-protein complexes (24). Indeed, at forces that promote protein disassembly, the force–extension curves of two consecutive ramps do not overlap anymore. In our assay, we detected a mild difference between the force–extension curves of two consecutive ramps at 6–7 pN for mtSSB^WT^-ssDNA, while the difference was a lot more pronounced for mtSSB^R107Q^-ssDNA, which indicates higher unbinding of mtSSB^R107Q^ at this mild tension. This further suggests that the interactions of mtSSB^R107Q^ with ssDNA are less stable than the interactions of mtSSB^WT^.

R107Q patient fibroblasts exhibit an important mtDNA depletion, suggesting a defect in mtDNA replication processes (14,32). This was confirmed by *in vitro* assays, which revealed that while the mutation does not impact mtDNA replication elongation, it impairs mtDNA replication initiation (8). Single-molecule experiments have shown that during mtDNA synthesis, the low site-size binding mode of mtSSB is favored at the replication fork (24). As our results indicate that mtSSB low binding mode is seemingly little affected by the R107Q mutation, this is thus consistent with the observation that mtDNA replication elongation is not affected in patients. On the contrary, we showed that mtSSB^R107Q^ ssDNA complexes in the wrapped mode are unstable, which may suggest a role for this binding mode in mtDNA replication initiation.

One hypothesis that has been proposed previously to explain the role of mtSSB disease-causing mutations is a lower stability of the tetramer. Previous *in-silico* modeling analysis of mtSSB^R107Q^ showed that shortening the side chain from Arg to Gln results in loss of the intra and inter dimer contacts which, considering these interactions occur in the four subunits, could lead to tetramer destabilization (14). However, using mass photometry (Figure 1), we observed that mtSSB^WT^ and mtSSB^R107Q^ were present in solution only in the tetramer form. Similarly, Gustafson *et al.*, using molecular dynamics simulation, predicted a very stable tetrameric structure for mtSSB^R107Q^ (37). The superposition of the ssDNA from *Ec*SSB structure (Figure 6) strongly suggests that the DNA locates right above Arg107 and also very close to Arg38, a mutation also involved in mitochondrial disease (14). Our structural model suggests that R107Q induces a change in the electrostatics precisely at this region, from positive to negative, plausibly creating a surface less prone to interact with the DNA, which could slow the transition to a stable, wrapped state. Overall, our results suggest that the R107Q mutation does not destabilize the formation of tetramers but instead affect the stable binding of mtSSB to ssDNA in a wrapped state, leading to a faster dissociation and a lower compaction ability. This is consistent with the phenotype observed in patients, where the mutation does not drastically compromise mtDNA maintenance but rather leads to adult-onset optic myopathy.

## Supplementary Material

gkae354_Supplemental_Files

## Data Availability

The data underlying this article will be shared on reasonable request to the corresponding author.

## Notes

Present address: Aleix Tarrés-Solé, ALBA Synchrotron, 08290 Cerdanyola del Vallès, Barcelona, Spain.
