# Supplementary material for: The mutation R107Q alters mtSSB ssDNA compaction ability and binding dynamics
Source: Nucleic Acids Res. 2024 Apr 14;52(10):5912–27. doi: 10.1093/nar/gkae354 (PMC11162770; doi:10.1093/nar/gkae354)
Supplement: gkae354_Supplemental_Files [file gkae354_supplemental_files.zip › Supplementary data_Martucci et al._revision_v110524.pdf]

SUPPLEMENTARY DATA

Supplementary figures

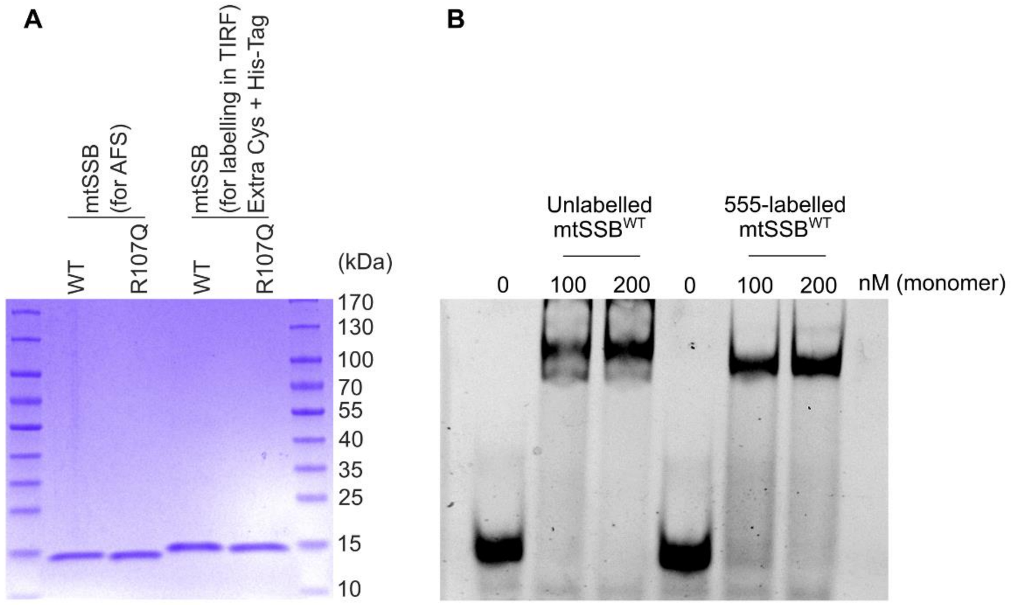

Figure S1. Quality control of the recombinant mtSSB proteins. **(A)** SDS-PAGE gel stained with Coomassie Blue showing the purity of the recombinant mtSSB proteins used for the AFS experiment (no tag) and the TIRF experiment (extra cysteine in C-ter for labelling purposes and His-tag in N-ter). **(B)** mtSSB binding to ssDNA as monitored by electromobility shift assay. Increasing unlabelled and 555-labelled mtSSB^{WT} proteins (extra cysteine in C-ter and His-tag in N-ter) were incubated at increasing concentrations (0, 100 and 200 nM, calculated as an mtSSB monomer) with 3.3 nM 22-nt oligonucleotide labelled with ATTO647N for 10 min at room temperature.

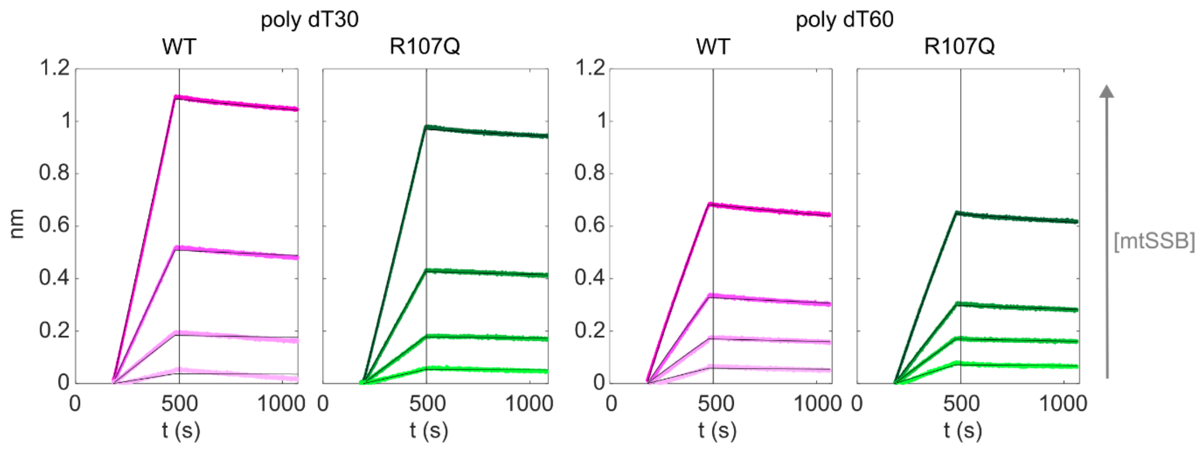

Figure S2. The affinity of $mtSSB^{WT}$ and $mtSSB^{R107Q}$ for ssDNA assessed by biolayer interferometry. Two ssDNA (poly-dT30 and poly-dT60) and four different mtSSB concentrations (0.625 nM, 1.5 nM, 2.5 nM and 5 nM) were used. The raw binding traces (green and magenta) were fitted with a 1:1 binding model (black curve).

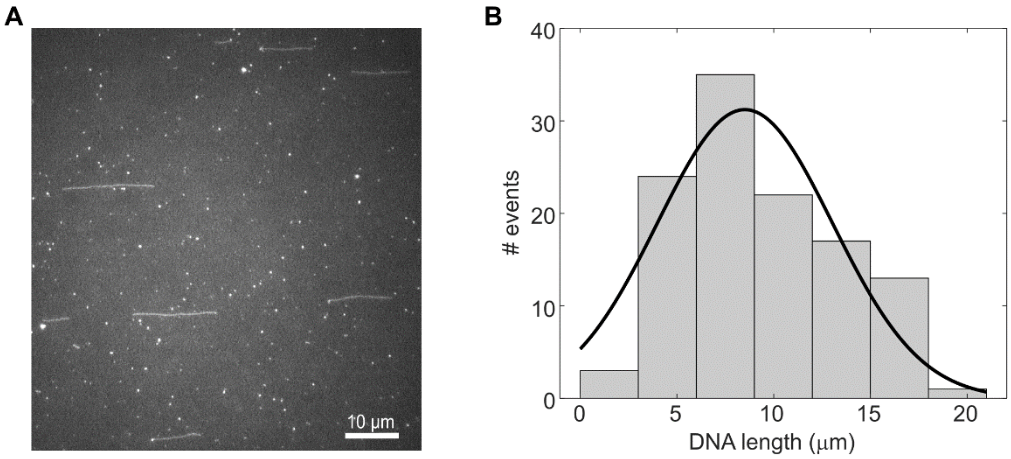

Figure S3. Determination of ssDNA molecules length under our flow conditions using SYBR Green II staining. **(A)** Typical image of a field of view showing several ssDNA molecules stained with SYBR Green II and stretched under a constant flow. **(B)** Distribution of ssDNA lengths. A Gaussian fit to the histogram yielded an average length of ~8.5 µm (n=115).

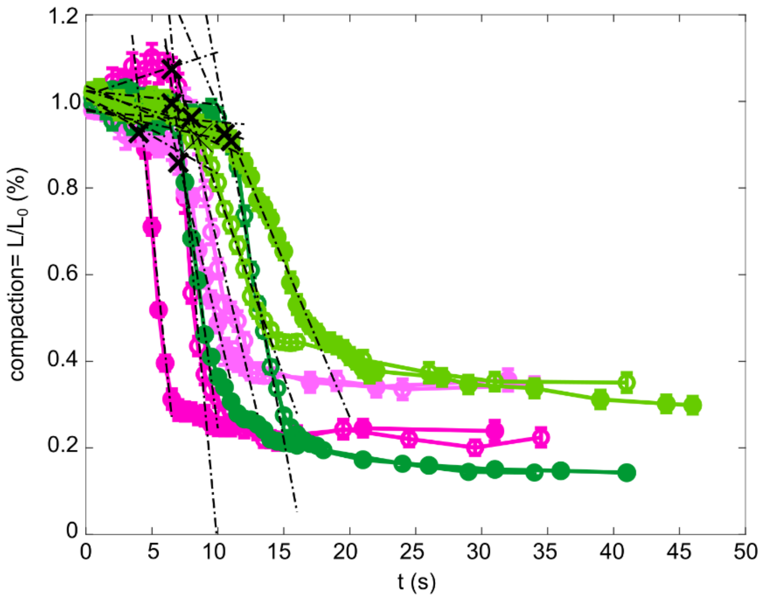

Figure S4. Determination of t0*. The time at which the salt begins to have an effect t0* is marked with a cross and is inferred from the intercept of a linear fit to the first (constant) part and a second linear fit to the first five points of the fast decaying part of the normalized compaction curve (dark green: $mtSSB^{WT}$ at 400 mM salt, light green: $mtSSB^{WT}$ at 100 mM salt, dark pink: $mtSSB^{R107Q}$ at 400 mM salt, light pink: $mtSSB^{R107Q}$ at 100 mM salt). Open and closed symbols of the same color represent 2 biological replicates.

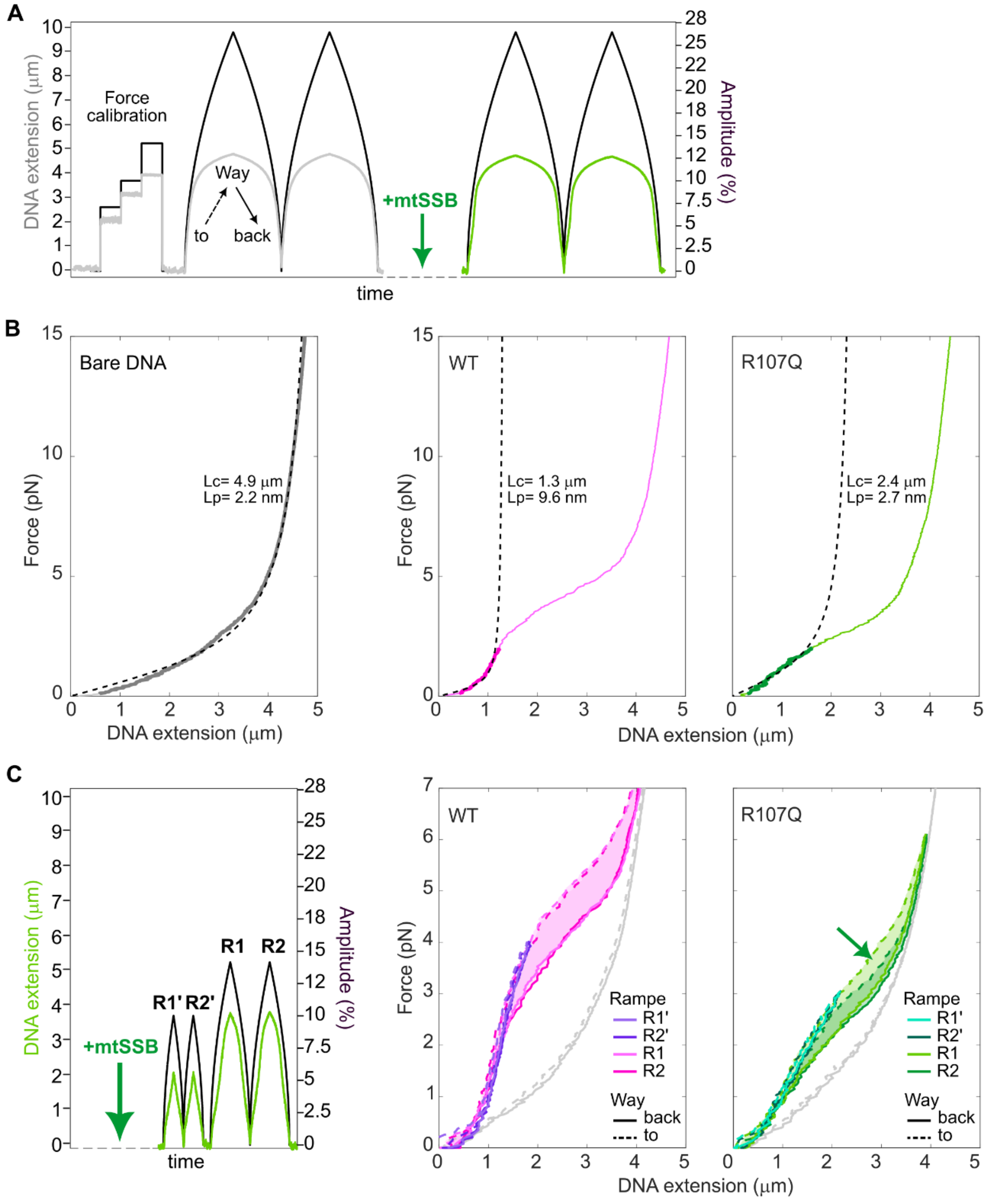

Figure S5. The R107Q mutation alters the ssDNA-binding properties of mtSSB. **(A)** Schematic of a typical AFS experiment. Following force calibration on bare ssDNA, force stretching (way to) and relaxation (way back) of the bare DNA is performed using two successive identical force ramps. The force ramps are then repeated after addition of the mtSSB protein. **(B)** Experimental force-retraction curves of representative DNA molecules (from the second ramps in (A)) of bare ssDNA (gray), or ssDNA coated with $mtSSB^{WT}$ (magenta) or $mtSSB^{R107Q}$ (green), and corresponding fits to the eFJC model (black dashed lines) to estimate the Lc and Lp parameters. Fitting range for bare DNA curve: 0.1-15 pN and for mtSSB-coated DNA: 0.1-2 pN. **(C)** A schematic of the force-dependence AFS experiment is shown on the left. After addition of the mtSSB protein, successive force ramps with low amplitudes are applied, first with 3-4 pN (R'1 and R'2) and then with 6-7 pN (R1 and R2). The corresponding force-distance extension (dashed lines) and retraction (solid lines) curves are shown on the right for two representative DNA tethers coated with mtSSB WT or mutant.

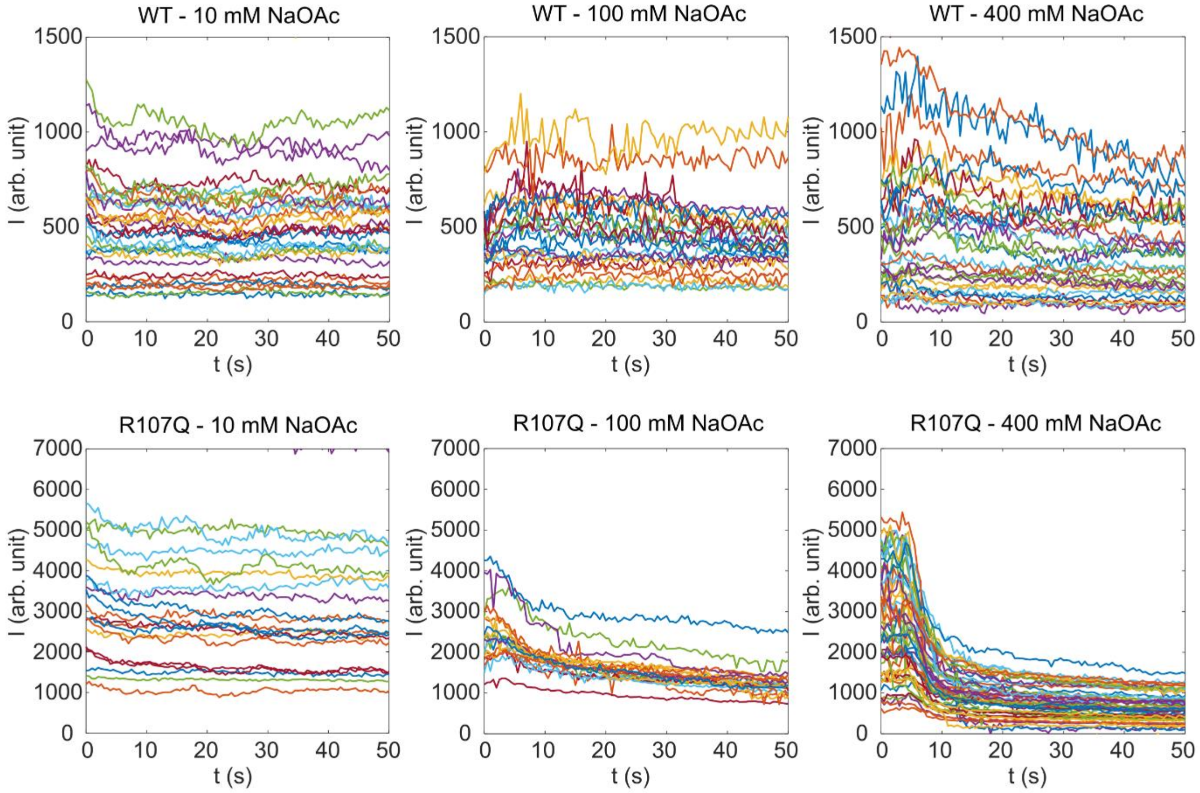

Figure S6. Total fluorescence intensity of mtSSB-coated-ssDNA molecules over time. To quantify total fluorescence intensity, a region of interest (ROI) that encompasses the entire fluorescent intensity along the DNA was selected at each time point and a local background subtraction correction was performed.

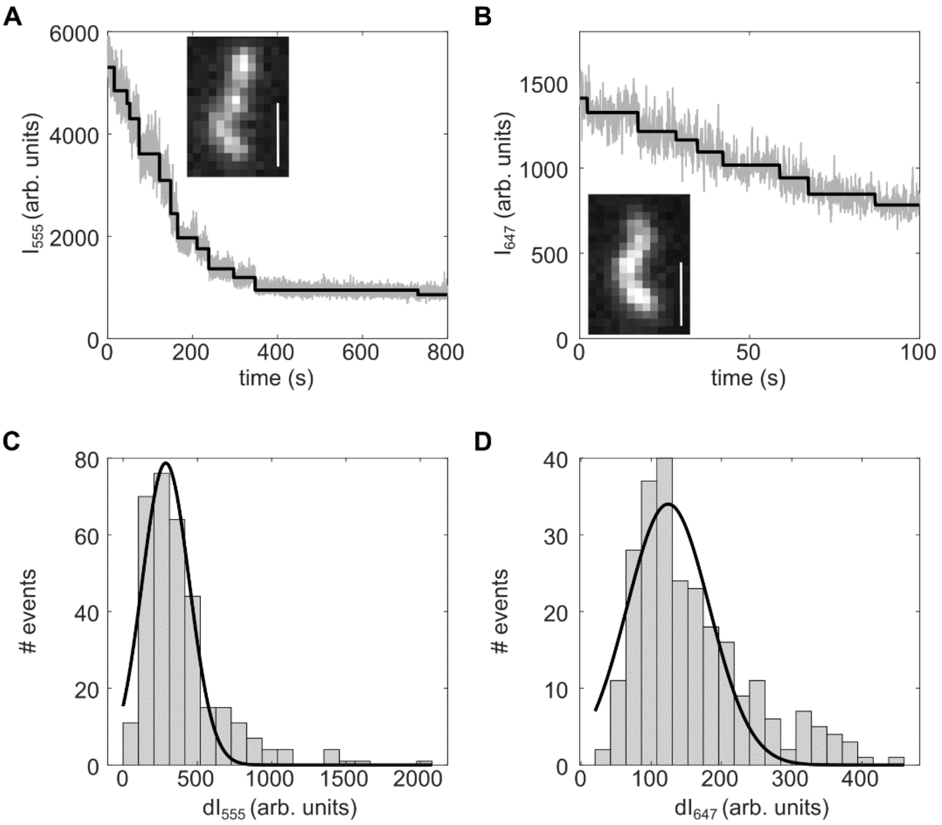

Figure S7. Quantification of the intensity of a mtSSB monomer. Fluorescence signal of a mtSSB^{Alexa555} **(A)** and mtSSB^{Alexa647} **(B)** complex as a function of time, showing single-step photobleaching events. Assays were performed using 100 ms exposure time and 4 mW and 11,4 mW laser power for 532 nm and 638 nm laser, respectively. The intensity of SSB coated-ssDNA was retrieved from kymographs and analyzed using AutoStepFinder v2.1.0 from Loeff-Kerssemakers et al (52), yielding the intensity loss during a photobleaching step, which is shown in the intensity histogram of single mtSSB^{Alexa555} and mtSSB^{Alexa647} complexes represented in **(C)** and **(D)**, respectively. The intensity distribution was fitted with a Gaussian, yielding a mean fluorescence value (dI) of 287 ± 51 a.u. (mean ± SE) (N= 328) for a single mtSSB^{Alexa555} complex and 124 ± 26 a.u. (mean ± SE) (N= 294) for a single mtSSB^{Alexa647}.

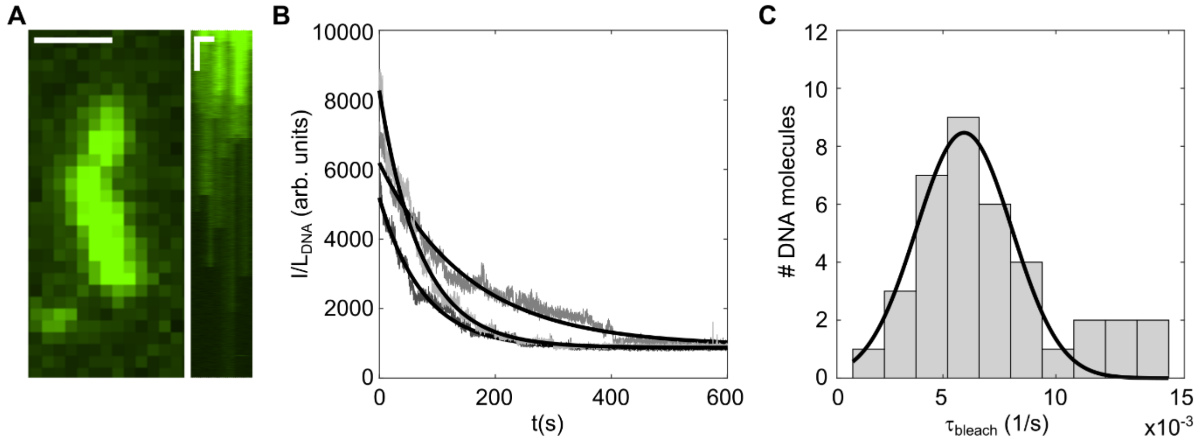

Figure S8. Photobleaching rate of mtSSB$^{WT\text{-}Alexa555}$. **(A)** Typical fluorescence image (left) and kymograph (right) of a mtSSB$^{WT\text{-}Alexa555}$-ssDNA nucleoprotein under continuous illumination, with an exposure time of 100 ms and a laser power of 4 mW. Scale bars : 1 µm (horizontal) and 2 min (vertical). **(B)** Fluorescence intensity over time from the kymograph displayed in (A). **(C)** From exponential fit to such data from a Gaussian fit to the obtained histogram we obtained a mean photobleaching rate with a standard deviation; $k_{\{bleaching\}}$= 5.9e-3 +/- 1.3 e-3 1/s (N=38). The dissociation experiments were performed with a factor 2 less laser power and with a delay between frames.

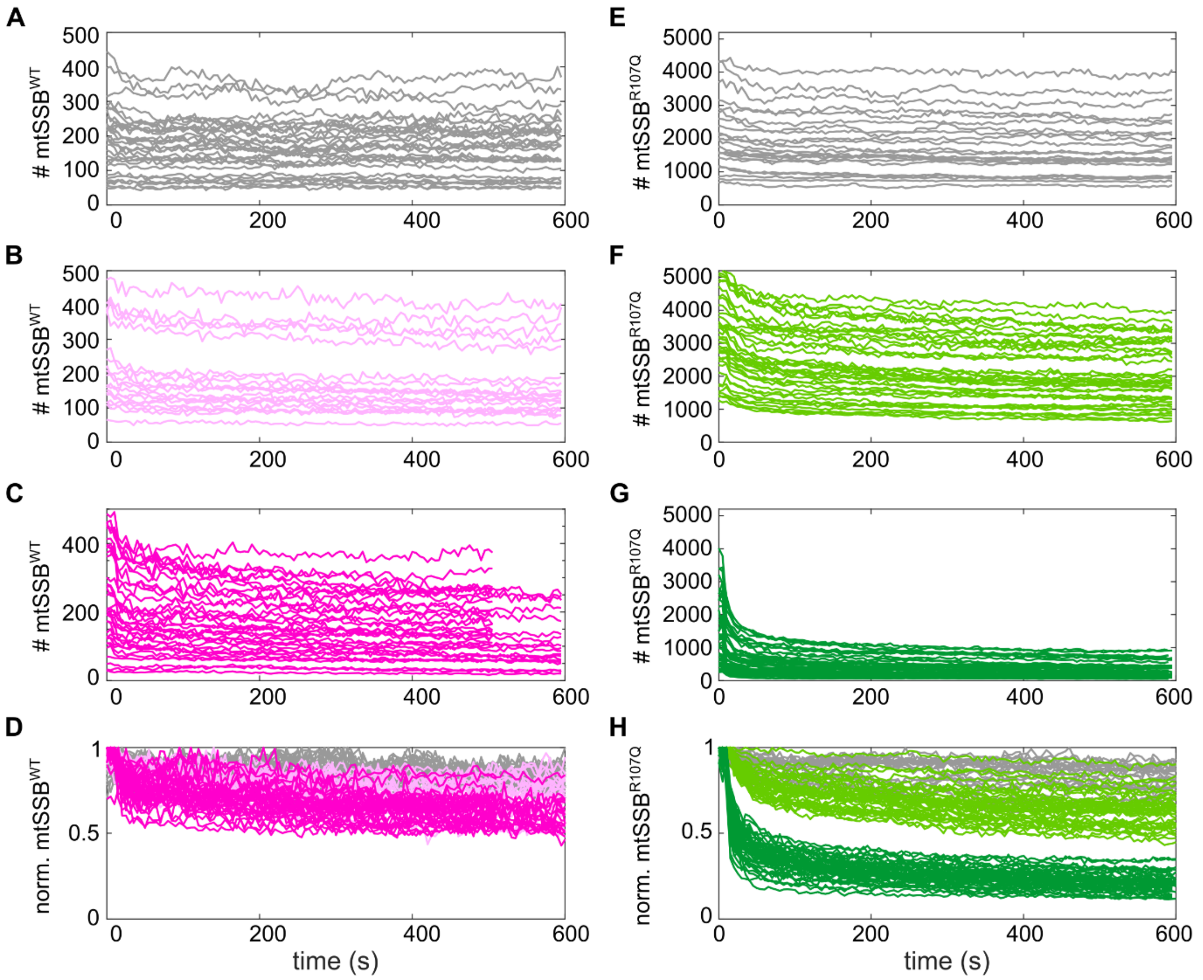

Figure S9. Number of mtSSB^{WT} or mtSSB^{R107Q} monomers bound to ssDNA over time at different salt concentrations. **(A)** WT, 10 mM NaOAc, **(B)** WT,100 mM NaOAc, **(C)** WT, 400 mM NaOAc, **(D)** WT, all traces superimposed, **(E)** R107Q, 10 mM NaOAc, **(F)** R107Q, 100 mM NaOAc, **(G)** R107Q, 400 mM NaOAc, **(H)** R107Q, all traces superimposed. For each experimental condition, only ssDNA molecules larger than 1 µm were considered, which corresponds to ~30-40 ssDNA molecules per condition.

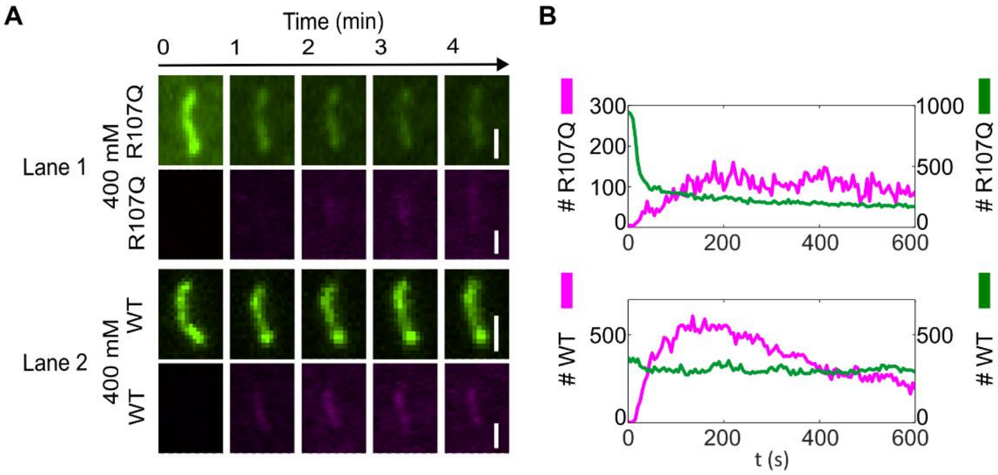

Figure S10. Real time competition assays. **(A)** Fluorescence images of nucleoprotein complexes over time. The upper line of each configuration shows mtSSB$^{R107Q\text{-}Alexa555}$ (lane 1) or mtSSB$^{WT\text{-}Alexa555}$-ssDNA (lane 2) complexes stretched at the surface of the flow-cell. The lower line shows mtSSB$^{R107Q\text{-}Alexa647}$ or mtSSB$^{WT\text{-}Alexa647}$ that is added to the flow-cell in the presence of salt. **(B)** Intensity measurement of mean intensities of ssDNA-mtSSB complexes as a function of time for each competition assay.

Supplementary movies

Movie S1: This movie shows ssDNA compaction by $mtSSB^{WT\text{-}Alexa555}$ after addition of 100 mM NaOAc (corresponds to **Fig 2**).

Movie S2: This movie shows ssDNA compaction by $mtSSB^{R107Q\text{-}Alexa555}$ after addition of 100 mM NaOAc (corresponds to **Fig 2**).

Movie S3: This movie shows the competition between $mtSSB^{R107Q}$ (green) bound to ssDNA stretched at the surface of the flow-cell and free $mtSSB^{WT}$ (red) added to the flow-cell (corresponds to **Fig 5D lane 2**).

Movie S4: This movie shows the competition between $mtSSB^{WT}$ (green) bound to ssDNA stretched at the surface of the flow-cell and free $mtSSB^{R107Q}$ (red) added to the flow-cell (corresponds to **Fig 5D lane 3**).

Supplementary reference

52. Loeff, L., Kerssemakers, J.W.J., Joo, C. and Dekker, C. (2021): A fast and automated step detection method for single-molecule analysis. *Patterns (N Y)*, 2, 100256.
